# Dietary N-6 Polyunsaturated Fatty Acid Intake and Brain Health in Middle-Aged and Elderly Adults

**DOI:** 10.3390/nu16244272

**Published:** 2024-12-11

**Authors:** Jiawei Gu, Yujia Bao, Yongxuan Li, Li Hua, Xiaobei Deng, Yuzheng Zhang, Xiaojun Zhu, Jinjun Ran

**Affiliations:** 1 School of Public Health, Shanghai Jiao Tong University School of Medicine, Shanghai 200025, China; jw.gu0312@sjtu.edu.cn (J.G.); bubble-y@sjtu.edu.cn (Y.B.); melody321@sjtu.edu.cn (Y.L.); seyhuali@shsmu.edu.cn (L.H.); dengxiaobei@sjtu.edu.cn (X.D.); 2 National Center for Mental Health, Beijing 100121, China; zhangyuzheng.jenny@gmail.com; 3 School of Mathematics and Physics, Xi’an Jiaotong-Liverpool University, Suzhou 215123, China

**Keywords:** dietary factors, n-6 polyunsaturated fatty acids, neurodegenerative disease, gray matter, white matter, genetic risk

## Abstract

Background: Dietary intake of polyunsaturated fatty acids (PUFA) plays a significant role in the onset and progression of neurodegenerative diseases. Since the neuroprotective effects of n-3 PUFA have been widely validated, the role of n-6 PUFA remains debated, with their underlying mechanisms still not fully understood. Methods: In this study, 169,295 participants from the UK Biobank were included to analyze the associations between dietary n-6 PUFA intake and neurodegenerative diseases using Cox regression models with full adjustments for potential confounders. In addition, multiple linear regression models were utilized to estimate the impact of n-6 PUFA intake on brain imaging phenotypes. Results: Results indicated that low dietary n-6 PUFA intake was associated with increased risks of incident dementia (hazard ratio [95% confidence interval] = 1.30 [1.13, 1.49]), Parkinson’s disease (1.42 [1.16, 1.74]), and multiple sclerosis (1.65 [1.03, 2.65]). Moreover, the low intake was linked to diminished volumes of various brain structures, including the hippocampus (*β* [95% confidence interval] = −0.061 [−0.098, −0.025]), thalamus (−0.071 [−0.105, −0.037]), and others. White matter integrity was also found to be compromised in individuals with low n-6 PUFA intake. Conclusions: These findings enhanced our understanding of how dietary n-6 PUFA intake might affect neurological health, thereby providing epidemiological evidence for future clinical and public health interventions.

## 1. Introduction

With the rapid global increase in aging populations, the burden of neurodegenerative diseases has become a major public health challenge, significantly affecting individual well-being and societal resources [1,2]. Conditions including dementia (DEM) and Parkinson’s disease (PD) are escalating in prevalence among others [3], necessitating a greater focus on modifiable risk factors, particularly dietary components, which are increasingly recognized as critical determinants in the onset and progression of these neurodegenerative disorders [4,5]. The connection between diet and brain health has gained substantial interest in recent years, especially as research continues to reveal the significant impact of specific nutrients and dietary patterns on cognitive function among the elderly population [6]. Given the growing body of evidence linking dietary factors to brain health, including studies exploring their influence on brain structure, there is an urgent need for targeted nutritional interventions to mitigate the increasing global burden of neurodegenerative diseases.

Among dietary factors, polyunsaturated fatty acids (PUFAs) have been extensively recognized for their critical roles in maintaining brain health, with omega-6 (n-6) and omega-3 (n-3) fatty acids drawing particular attention. These essential nutrients, predominantly derived from dietary sources, are integral to neuronal membrane composition and functionality, playing vital roles in neurophysiological processes [7,8]. Although the neuroprotective properties of n-3 PUFA have been well-established, the implications of dietary n-6 PUFA remain inadequately explored, with findings that are both limited and inconsistent [9]. Some studies have suggested that elevated n-6 PUFA intake is associated with heightened neuroinflammation and an increased risk of DEM [10]. In contrast, other findings indicated that insufficient n-6 PUFA consumption may also impair neuronal function and exacerbate cognitive decline, underscoring the complex and context-dependent nature of its effects [11,12]. These discrepancies may, in part, stem from methodological limitations in existing studies, including small sample sizes, cross-sectional designs, heterogeneous dietary patterns across populations, and others [9,13]. Furthermore, while mechanistic insights have been partially derived from animal models, the biological pathways through which n-6 PUFA influences neurodegeneration remain insufficiently characterized [14,15,16]. Compounding these challenges is the scarcity of robust epidemiological evidence, which further hinders efforts to reconcile conflicting findings. These limitations collectively underscore the pressing need for large-scale, longitudinal cohort studies to systematically clarify the role of n-6 PUFA in neurodegeneration and cognitive health.

Our study seeks to fill the gap in understanding the relationship between dietary n-6 PUFA intake and neurodegenerative diseases, including DEM, PD, and multiple sclerosis (MS), as well as brain structural traits, including alterations in gray matter (GM) volumes and white matter (WM) microstructural integrity. To overcome the limitations of previous studies, we utilized a large-scale cohort with robust dietary assessments, detailed neuroimaging data, and extensive follow-up periods. Moreover, subgroup analyses stratified by genetic risks and demographic factors were conducted to capture context-specific effects and refine the interpretation of findings. By employing these comprehensive approaches, our findings are expected to offer critical insights into the nuanced roles of dietary fats in neurological health, laying a foundation for targeted clinical strategies and public health interventions aimed at mitigating the burden of neurodegenerative diseases.

## 2. Materials and Methods

### 2.1. Study Population

The UK Biobank is an ongoing prospective cohort study that recruited approximately 500,000 individuals aged 37 to 73 during the baseline period between 2006 and 2010. Recruitment was conducted at 22 assessment centers distributed across England, Scotland, and Wales. The centers encompassed diverse environments to capture a broad range of socioeconomic factors and health variation, utilizing touchscreen questionnaires, physical assessments, biological samples, genetic sequencing, and other measurements to provide a comprehensive dataset for health research. Participants were monitored for disease occurrence through links to national health records. The North West Multi-Center Research Ethics Committee has granted approval for the UK Biobank, and all participants provided informed consent through completed forms (UK Biobank application number: 99001 [31 January 2023 to 31 January 2026]). The study protocol of the UK Biobank can be found online (www.ukbiobank.ac.uk, accessed on 8 October 2024). Participants lacking exposure or outcome data, specific covariates, or with neurodegenerative diseases at baseline were excluded from the study. Moreover, participants with high levels of missing data, excessive heterozygosity, or discrepancies between genetic sex and self-reported sex were also excluded. Finally, a total of 169,295 participants were included in this study, including 20,504 with available MRI data. Our study followed the Strengthening the Reporting of Observational Studies in Strengthening the Reporting of Observational Studies in Epidemiology guidelines.

### 2.2. Exposure Assessment

In the UK Biobank, dietary intake was assessed using the Oxford WebQ, a validated online dietary assessment tool facilitated by interviewer support [17]. Previous research has shown that this method provides reliable estimations of nutrient consumption. From 2009 to 2021, participants were asked to complete the questionnaire up to five times, detailing their consumption of 206 food items and 32 beverages within the past 24 h. For this analysis, individuals who participated in at least one dietary assessment were included, and mean nutrient intake values were calculated across the assessments to minimize measurement bias. The focus of this analysis was on n-6 polyunsaturated fatty acid (PUFA) intake, an essential component of the diet with known implications for inflammation and various health outcomes, including neurological disorders [18]. Due to the absence of established dietary guidelines for n-6 PUFA intake, participants’ intake levels were dichotomized into low-intake and high-intake groups based on the median intake (cut-off value: 10.15 g/d) with the high-intake group serving as the reference group.

### 2.3. Outcome

The primary outcomes included a broad range of neurodegenerative diseases, including DEM, PD, and MS. Hospital inpatient diagnoses from the UK Biobank were retrieved through linkages with national health registers. These cases were coded using the International Classification of Diseases, 9th (ICD-9) and 10th editions (ICD-10). Detailed definitions of neurodegenerative diseases in the UK Biobank are provided in Supplementary Table S1. Participants were followed from the date of their baseline assessment to the earliest of the following endpoints: diagnosis of any neurodegenerative disease, death, the last available date in either primary care data or hospital inpatient data, or 19 December 2022, whichever occurred first.

The secondary outcomes of this study were centered on changes in brain structure, specifically alterations in GM and WM. Structural imaging and diffusion data were processed by the UK Biobank team and made available to approved researchers as imaging-derived phenotypes (IDPs). MRI data were obtained from the UK Biobank beginning in 2014, and conducted at specialized assessment centers equipped with Siemens Skyra 3T scanners and 32-channel head coils [19]. The scanning protocol and sequence parameters have been previously documented and are publicly accessible. GM phenotypes were extracted from high-resolution T1-weighted images processed with FreeSurfer software, version 6.0.0. The quality of the FreeSurfer outputs was evaluated through the Qoala-T framework, and manual verifications were conducted for results approaching quality thresholds. Outputs that did not meet the established quality standards were excluded from the analysis. This study involved quantifying the volumes of various functional GM regions, including data for 33 cortical regions (UKB data field 192), as well as 7 subcortical regions (UKB data field 1102) using FreeSurfer’s aseg tool. WM integrity was assessed using diffusion-weighted imaging (DWI) data processed by the UK Biobank. Key diffusion tensor imaging (DTI) metrics, including fractional anisotropy (FA), mean diffusivity (MD), and intra-cellular volume fraction (ICVF), were extracted from 15 WM tracts (UKB data field 135) [20]. These indices provide insights into WM microstructural integrity, where FA indicates the degree of directionality in water diffusion, MD represents overall diffusivity, and ICVF reflects intracellular space and tissue density. All continuous measures of brain structure, encompassing both GM volumes and WM integrity metrics were normalized to facilitate interpretation and comparison across individuals. Detailed information on the brain structure metrics can be found in Supplementary Table S2.

### 2.4. Covariates

To mitigate the influence of confounding variables on neurodegenerative outcomes and n-6 PUFA intake, various covariates were integrated into the statistical models (Supplementary Table S3). These covariates encompassed age, sex, the index of multiple deprivation (IMD), waist-hip ratio (WHR), healthy lifestyles (never smoking, no heavy alcohol intake, healthy sleep pattern, healthy diet, and regular physical activity). Additionally, systolic and diastolic blood pressure (SBP, DBP) and critical metabolic biomarkers, including glucose, glycated hemoglobin (HbA1c), triglycerides (TG), and low-density lipoprotein cholesterol (LDL) were also considered. To ensure the robustness of the analysis and account for potential confounding effects, dietary intake of n-3 PUFA and the n-3/n-6 PUFA ratio were also included as covariates in the statistical models.

The IMD was utilized to assess social deprivation at the neighborhood level, encompassing seven distinct domains, which include health, education, income, employment, crime, barriers to housing and services, and living environment [21]. The WHR was calculated based on waist and hip circumference measurements collected during the initial assessment, and it was classified into two categories: poor (≥0.9 for men and ≥0.85 for women) and ideal (<0.9 for men and <0.85 for women) [22]. Never smoking was defined for individuals who reported no previous or current smoking at baseline [23]. No heavy alcohol intake was defined as the average daily intake ≤16 g of pure alcohol (2 units of alcohol) for both men and women [24]. Regular physical activity was assessed according to the American Heart Association’s guidelines, which recommend engaging in a minimum of 150 min of moderate-intensity exercise or 75 min of vigorous-intensity exercise (or an equivalent combination) per week [25]. A healthy diet was characterized by the consumption of at least four out of seven food groups that are commonly recommended as dietary priorities for cardiometabolic health. The specific frequencies for each component of a healthy diet are as follows: ≥3 servings/day for fruit; ≥3 servings/day for vegetables; ≥2 servings/week for fish; ≤1 serving/week processed meats; ≤1.5 servings/week for unprocessed red meats; ≥3 servings/day for whole grains; ≤1.5 servings/day for refined grains [23]. A healthy sleep pattern was characterized by meeting at least four out of five criteria for healthy sleep behaviors, which included: an early chronotype; sleeping 7 to 8 h per day; experiencing insomnia rarely or never; not self-reporting snoring; and dozing off during the day rarely or never [26]. 

### 2.5. Polygenic Risk Score (PRS)

PRS showed the association between genotype and risk of incident DEM, PD, and MS by score points, and was composed of single nucleotide polymorphisms (SNPs). The PRS for DEM, PD, and MS was calculated by using 83, 44, and 155 SNPs respectively (Supplementary Tables S4–S6), all of which passed quality control, based on a previous meta-analysis of genome-wide association studies. A weighted method was used to calculate the PRS. Each SNP was recoded as 0, 1, or 2 according to the number of risk alleles, and each SNP was multiplied by a weighted risk estimate (*β* coefficient) on targeted disease obtained from the previous genome-wide association study. The formula for calculation is

$$\mathrm{PRS}=\sum_{i=1}^{j}\beta$$

*j* is the total number of SNPs. This represents the effect allele of the number × SNP. The higher levels of PRS represent the higher genetic susceptibility to our targeted diseases. In this study, the PRS was categorized into low (the lowest tertile), intermediate (the middle tertile), and high (the highest tertile) risk.

### 2.6. Statistical Analysis

The baseline characteristics of the study participants were summarized as medians (interquartile range [IQR]) for continuous variables and counts (percentages) for categorical variables. After assuring that the proportional hazard assumption was met through the Scaled Schoenfeld Residual, cox proportional hazard regression models were utilized to assess the associations between dietary n-6 PUFA intake and the incident risks of neurodegenerative diseases. Missing values were deleted from the analyses. Tertiary model strategies were adopted, Model 1 adjusted baseline characteristics including age, sex, and IMD. Model 2 additionally adjusted WHR and healthy lifestyles (never drinking, no heavy alcohol intake, healthy sleep pattern, healthy diet, and regular physical activity). Model 3 additionally adjusted SBP, DBP, metabolic biomarkers (glucose, HbA1c, TG, LDL), dietary n-3 PUFA intake and dietary n-3/n-6 PUFA intake ratio based on Model 2. Hazard ratios (HRs) and corresponding 95% confidence intervals (CIs) were calculated using Cox proportional hazards regression models. To enhance the robustness of the study framework and ensure the reliability of the findings, the association between dietary n-3 PUFA intake and neurodegenerative diseases was examined as a positive control, considering its well-established neuroprotective effects in dementia and Parkinson’s disease [27,28,29,30,31]. To further identify the potential modifiers of the associations above, a series of stratified analyses were performed by age (45–59/≥60 [60–64/≥65]), sex (female/male), IMD (low/high), WHR (poor/ideal), prevalent disease (hypertension, diabetes) (yes/no), genetic risks (low/moderate/high), physical frailty (yes/no), and telomere length (short/long). A test of the interaction between dietary n-6 PUFA intake and each term was conducted in the model by including a multiplicative term.

For subsequent neuroimaging analyses, multiple linear regression models were utilized to assess the associations between dietary n-6 PUFA intake and brain structural imaging phenotypes, involving the volumes of distinct GM regions and metrics of WM integrity, specifically FA, MD, and ICVF, with the same adjustment in Model 3. Stratified analysis by sex (female/male) was conducted to estimate the potential effect modification. Effect values were shown as the *β*s with corresponding 95% CIs, with statistical significance determined by *p* values.

Several sensitivity analyses were conducted to evaluate the robustness of our results. First, we restricted the cohort to individuals with White European ancestry, given that non-White participants represent only approximately 3% of the total dataset. Second, we controlled various environmental factors (proximity to roadways, noise pollution, nitrogen oxide, and fine particulate matter [PM2.5]) that could potentially influence the outcomes. Third, participants who had been diagnosed with a targeted disease event within the first two years of follow-up were excluded from the study. Fourth, in consideration of the impact of the COVID-19 outbreak on our findings, we advanced the deadline for follow-up to 31 December 2019 [23]. Finally, despite adjusting for IMD in the models, residual confoundings related to socioeconomic status may still persist. To address this, we further included income and education as covariates. The statistical significance was determined by a two-tailed *p* value of less than 0.05. All analyses were conducted using R software (version 4.2.2), survival for Cox proportional hazards regression, stats for logistic regression and other statistical analyses, and forestmodel for the creation of forest plots. 

## 3. Results

**Figure 1:**
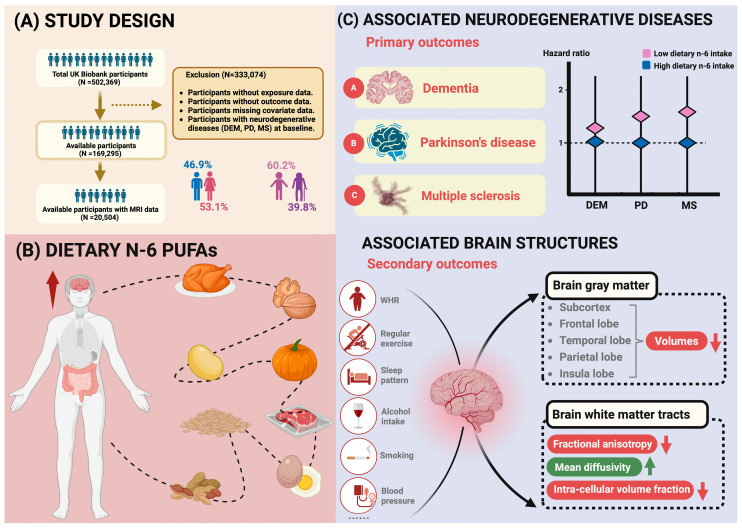
Study workflow. This study analyzed data from 169,295 UK Biobank participants, tracking outcomes for three neurodegenerative diseases, with brain structural alterations considered as secondary outcomes. (**A**) the study cohort, including population flowchart and baseline characteristics. (**B**) dietary intake of n-6 PUFAs. (**C**) Cox proportional hazards regression models were applied to examine associations between low n-6 PUFA intake and neurodegenerative diseases risk. Hazard ratios (HRs) and the corresponding 95% confidence intervals (CIs) were calculated in our analyses. Subgroup analyses were conducted stratified by age (<65/≥65), sex (male/female), waist-hip ratio (poor/ideal), and others. Further analyses were also utilized to estimate the relationships of PUFA intake with impairments of brain gray and white matter by multiple linear regression models.

Following the exclusion of 333,074 participants lacking data on exposure, outcome, and specific covariates, as well as those diagnosed with a specific outcome at baseline, a total of 169,295 participants were enrolled in this study (Figure 1). Among the participants, 61.5% were under the age of 65, 53.1% were female, 50.0% had high IMD, and 45.7% had poor WHR. Statistics of the baseline information for the individuals analyzed for associations between dietary n-6 PUFA intake and neurodegenerative diseases are shown in Table 1. In total, 155,840 (92.2%) reported no smoking, 111,983 (66.2%) reported no heavy alcohol intake, 91,404 (54.0%) engaged in regular physical activities, 95,932 (56.7%) had a healthy sleep pattern, 69,180 (40.9%) adhered to a healthy diet. Supplementary Table S7 provides data on neurodegenerative disease occurrences disaggregated by sex, shown as total person-years and incidence rates.

**Figure 2:**
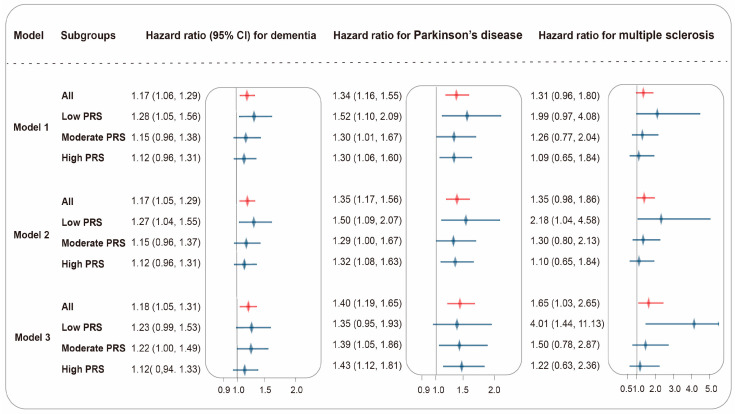
Associations between dietary n-6 PUFA intake and neurodegenerative diseases by genetic risks. A total of 169,295 participants were included to evaluate the relationship between low dietary n-6 PUFA intake and the incidence of dementia, Parkinson’s disease, and multiple sclerosis using the Cox proportional hazards regression model. Subgroup analyses were categorized into four groups: the overall population, low polygenic risk score (PRS), moderate PRS, and high PRS. Results for the overall population were presented in red, and those for the other subgroups were displayed in blue. Model 1 adjusted baseline characteristics including age, sex, and IMD. Model 2 further adjusted WHR and healthy lifestyle factors. Model 3 added adjustments for blood pressure, metabolic biomarkers, dietary n-3 PUFA intake and dietary n-3/n-6 PUFA intake ratio. Results were presented as HRs ± 95% CIs.

After full adjustments for age, sex, IMD, WHR, healthy lifestyle (never smoking, no heavy alcohol intake, healthy diet, healthy sleep pattern, and regular physical activity), metabolic biomarkers, dietary intake of n-3 PUFA and the n-3/n-6 PUFA ratio, low-level dietary n-6 PUFA intake was significantly associated with higher risks of cause-specific neurodegenerative diseases. The comparison of the dietary n-6 PUFA high-intake levels of the participants revealed that those with a low intake level exhibited elevated risks of DEM (HR [95% CI] = 1.30 [1.13, 1.49]), PD (1.42 [1.16, 1.74]), and MS (1.65 [1.03, 2.65]). The results of the positive control group were presented in Supplementary Table S8. The results of the subgroup analysis based on genetic risk were presented in Figure 2. In the age-stratified analysis, among participants aged ≥60, individuals in the 60–64 age group had a higher risk of experiencing dementia (1.72 [1.38, 2.14] for participants in 60–64, 1.08 [0.91, 1.29] for participants ≥65, P for interaction <0.001). Results demonstrated no significant difference in stratification analyses by sex, WHR, IMD, hypertension, diabetes, physical frailty, and telomere length (Table 2 and Table 3). 

**Figure 3:**
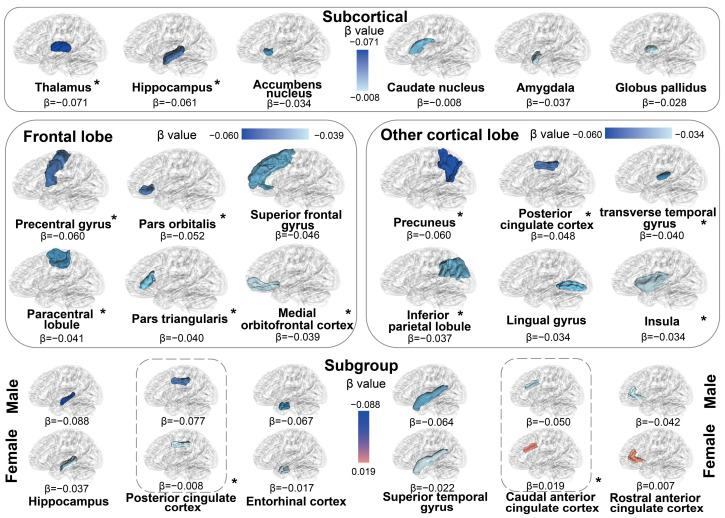
Associations between dietary n-6 PUFA intake and the volume of brain gray matter. The relationships between dietary n-6 PUFA intake and the volumes of the brain subcortex and cortex were assessed using multiple linear regression models among 20,506 participants with available MRI data from the UK Biobank. Results are presented as *β* values, with significance determined by *p* values. Furthermore, the results of stratified analyses by sex are presented at the bottom. Regions marked in red denote positive correlations, while those in blue denote negative correlations. The color from light to dark indicates that the absolute value of the effect value changes from small to large. The brain structure is in bold font and with an asterisk which indicates the association of statistical significance.

**Figure 4:**
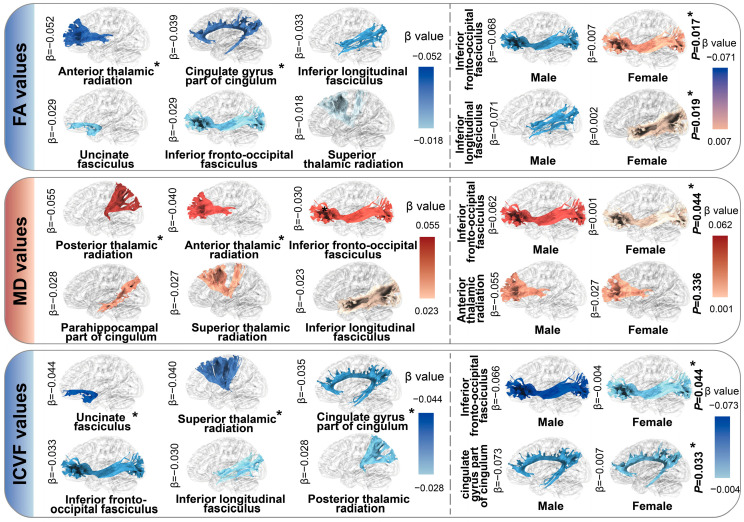
Associations between dietary n-6 PUFA intake and brain white matter integrity. The relationships between dietary n-6 PUFA intake and the integrity of white matter tracts were evaluated using multiple linear regression models among 20,504 participants with available MRI data from the UK Biobank. The fractional anisotropy (FA), mean diffusivity (MD), and intra-cellular volume fraction (ICVF) of white matter tracts were utilized as reference metrics. Results are presented as *β* values, with significance determined by *p* values. Furthermore, the results of stratified analyses by sex are presented on the right. Regions marked in red denote positive correlations, while those in blue denote negative correlations. The color from light to dark indicates that the absolute value of the effect value changes from small to large. The brain structure is in bold font and with an asterisk which indicates the association of statistical significance.

Our study further evaluated the relationships of dietary n-6 PUFA intake with brain structures. The descriptive statistics for brain phenotypes are demonstrated in Supplementary Table S9. The results of multiple linear regression analyses are displayed in Supplementary Tables S10 and S11. In terms of the volumes of GM, 3 out of 7 associations between dietary n-6 PUFA intake and subcortical regions, and 5 out of 33 associations with cortical structures remained significant after FDR correction. We observed that low-level dietary n-6 PUFA intake was negatively related to the atrophic volume of hippocampus (*β* [95% CI] = −0.061 [−0.098, −0.025]) and thalamus (−0.071 [−0.105, −0.037]). The shrunken cortical regions were also observed in participants with low-level n-6 PUFA intake, including paracentral lobule (−0.041 [−0.079, −0.004]), pars orbitalis (−0.052 [−0.088, −0.017]) and others (Figure 3. Figure 3 was generated using the BrainNet Viewer toolbox in MATLAB R2024b). Following the evaluation of GM relationships, we examined the associations between dietary n-6 PUFA intake and brain WM microstructure. The results from linear regression analyses of DTI metrics, including FA, MD, and ICVF. Notably, negative associations were identified for FA in the tract anterior thalamic radiation (−0.052 [−0.090, −0.014]), indicating reduced WM integrity with lower dietary n-6 PUFA intake. Conversely, positive associations for MD were observed in the following tracts: tract anterior thalamic radiation (0.040 [0.005, 0.075]) and tract posterior thalamic radiation (0.055 [0.019, 0.090]), suggesting increased diffusivity indicative of compromised microstructural integrity. Additionally, a significant negative association for ICVF was also found with estimated *β*s of (−0.040 [−0.078, −0.003]) for tract superior thalamic radiation, (−0.044 [−0.081, −0.006]) for tract uncinate fasciculus, reflecting decreased intracellular volume and tissue density (Figure 4. Figure 4 was generated using the BrainNet Viewer toolbox in MATLAB R2024b). In the sex-stratified subgroup analyses, the associations between low dietary n-6 intake and brain traits alterations were generally stronger in males compared to females, including changes in GM (nucleus accumbens: −0.050 [−0.010, 0.001] for males, 0.019 [−0.029, 0.067] for females, *P* for interaction = 0.029; postcentral gyrus: −0.077 [−0.125, −0.030], −0.008 [−0.054, 0.037], *P* for interaction = 0.020) and WM (FA of tract inferior fronto-occipital fasciculus: −0.068 [−0.118, −0.018], 0.007 [−0.041, 0.055], *P* for interaction = 0.017; MD of tract inferior fronto-occipital fasciculus: 0.062 [0.014, 0.110], 0.001 [−0.045, 0.048], *P* for interaction = 0.044; ICVF of tract inferior fronto-occipital fasciculus: −0.066 [−0.115, −0.016], −0.004 [−0.051, 0.043], *P* for interaction = 0.044, and others). Associations of dietary n-6 PUFA intake with brain gray and WM phenotypes were shown in Supplementary Tables S12 and S13. The results of the sensitivity analyses indicated that the main findings were robust (Supplementary Table S14).

## 4. Discussion

This study, conducted with a large cohort from the UK, examined the associations between dietary n-6 PUFA intake and neurodegenerative diseases as well as relevant brain structural traits. The findings indicate that low n-6 PUFA intake was associated with increased risks of DEM, PD, and MS, and with diminished GM volume in several brain regions, including the nucleus accumbens, hippocampus, and thalamus, as well as cortical areas such as the frontal, temporal, and parietal lobes. Additionally, low n-6 PUFA intake was associated with reduced WM integrity, evidenced by changes in FA, MD, and ICVF metrics. These findings underscore the importance of dietary interventions in the context of rapid population aging, offering valuable insights into the mechanisms that drive the progression of neurodegenerative diseases.

As dietary and health concerns gained increasing attention, scientists gradually recognized the significant influence of diet on disease development. Changes in modern dietary patterns, particularly the regional variability in n-6 intake, sparked discussions regarding whether the intake of PUFAs met health recommendations. A systematic review of 53 studies from 17 European countries suggested that, while 52% of these countries met the recommended levels of linoleic acid (LA), significant deficiencies remained among specific groups, including breastfeeding women, adolescents, and the elderly [8]. These regional and demographic disparities led researchers to focus more on the role of n-6 PUFAs in certain diseases, further exploring the potential association between insufficient or excessive intake and disease risk. Early studies rarely considered the negative impacts of n-6 PUFA, focusing instead on its potential health benefits. However, with increasing knowledge of inflammatory mechanisms and their effects on the nervous system [5,9], research shifted toward examining the complex relationship between n-6 PUFA intake and disease development. A few but limited studies suggested that higher n-6 PUFA intake might benefit the nervous system. For example, early epidemiological investigations reported that higher n-6 PUFA intake was associated with slower cognitive decline, suggesting a potential neuroprotective role [32]. A large prospective study found that higher n-6 PUFA intake was linked to a lower incidence of DEM [33]. Moreover, studies on MS and PD also indicated that higher n-6 intake might reduce the incidence and slow disease progression [34,35]. However, the previous findings faced increasing scrutiny. Studies suggested that excessive n-6 PUFA intake might exacerbate neurodegenerative diseases by triggering inflammatory responses [36,37]. While certain studies showed a possible association between high n-6 intake and elevated disease risk, many others failed to produce significant findings [6]. These discrepancies could be attributed to the limitation of the study design, insufficiency of sample size, and differentiation in the follow-up period [11,38]. This ongoing uncertainty prompted further exploration of the underlying mechanisms, particularly in brain structure. Early research demonstrated a potential protective role of n-6 PUFA in maintaining GM volume [39]. Recent research evidenced that lower n-6 PUFA intake might be associated with an increased risk of neurodegenerative diseases and significant structural damage in the brain, especially in WM tracts [33]. Varied results, thus, underscored the complexity of dietary n-6 PUFAs in the context of neurological health and highlighted the need for further investigation. This study aimed to investigate the relationship of dietary n-6 PUFA intake with neurodegenerative diseases and brain structural impairments, thereby complementing existing research and improving the reliability of the findings.

Our findings revealed significant associations between low dietary n-6 PUFA intake and elevated risks of DEM, PD, and MS, with increased risks of 30%, 42%, and 65%, respectively. These results underscore the pivotal role of n-6 PUFAs in neuroprotection, aligning with prior studies that emphasize their anti-inflammatory and neuronal maintenance functions [40,41]. Mechanistically, n-6 PUFAs contribute to the biosynthesis of prostaglandins and leukotrienes, key regulators of inflammatory responses and neural homeostasis [42,43]. Animal studies have further demonstrated that diets deficient in n-6 PUFAs exacerbate microglial activation and oxidative stress, accelerating neuronal damage [44,45]. Such findings suggest that the chronic low-grade inflammation (LGI) observed in low n-6 PUFA intake may serve as a critical pathway through which dietary inadequacies contribute to neurodegeneration. Nevertheless, while these mechanisms offer plausible explanations, inconsistencies in epidemiological studies highlight the need for more controlled trials and mechanistic studies to validate these pathways and inform age-specific dietary interventions. 

In the age-stratified analysis, the elevated risk observed in individuals aged 60–64 underscored the importance of prioritizing this age group in dementia prevention efforts. Targeted interventions, including lifestyle modifications and early cognitive screening, may be particularly effective in this critical window, potentially reducing the risk of dementia progression and improving long-term cognitive outcomes. We also employed PRS to demonstrate the impact of varying genetic risk levels on disease susceptibility. The results demonstrated a clear trend in MS, where lower PRS levels were associated with a higher risk of the disease (4.01 [1.44, 11.13] for the low-PRS group, 1.50 [0.78, 2.87] for the moderate-PRS group, and 1.22 [0.63, 2.36] for high-PRS group). This indicated that individuals with a lower genetic predisposition to MS may be more susceptible to the detrimental effects of low-level dietary n-6 PUFA intake. The results could be explained by that people’s lower susceptibility to illness lead them to underestimate the impact of lifestyle factors, subsequently increasing their risk of disease. Furthermore, such individuals may exhibit a lack of vigilance in health management, preventing effective control of potential risks. Our findings emphasized the potential importance of targeting modifiable lifestyle factors, such as diet, within populations exhibiting low genetic risk, where the effects of such interventions could be more pronounced. The potential for personalized dietary interventions offers a new perspective for reducing disease incidence in high-risk populations and aligns with the principles of precision medicine, emphasizing the integration of genomic risk with nutritional intake [44]. As our understanding of the interplay between genetics and dietary patterns deepens, personalized nutrition strategies may emerge as essential tools for the prevention of neurodegenerative diseases.

In addition to the increased risk of neurodegenerative diseases, low dietary intake of n-6 PUFAs was associated with significant structural brain alterations, including atrophy in the hippocampus, nucleus accumbens, and thalamus—regions critical for cognitive and emotional regulation. These findings are consistent with experimental models demonstrating that n-6 PUFA deficiency impairs hippocampal neurogenesis and disrupts synaptic plasticity, key processes essential for maintaining neural integrity [46,47,48]. Moreover, the observed reductions in FA and ICVF, along with increases in MD within white matter tracts such as the thalamic radiation, mirror results from animal studies linking lipid dysregulation to compromised axonal integrity and impaired myelin maintenance [49,50]. Such microstructural changes are likely exacerbated by chronic LGI, mediated through disruptions in lipid metabolism and the release of pro-inflammatory cytokines, including C-reactive protein and TNF-α [51,52]. These inflammatory mechanisms are known to contribute to neuronal damage and white matter disconnection, further accelerating cognitive decline. Collectively, these findings underscore the broader neurobiological impact of insufficient n-6 PUFA intake, extending its role from functional impairments to structural damage, and emphasize the critical importance of maintaining adequate dietary n-6 PUFA levels to support brain health.

Our study carried significant public health implications, providing valuable epidemiological evidence regarding the role of dietary n-6 PUFA intake in neurodegenerative diseases and associated brain structural changes. By employing a large sample size cohort, we systematically examined the associations between n-6 PUFA intake and various neurodegenerative conditions. Moreover, our analysis, which incorporated subgroups including sex, age, genetic risk, and others, allowed us to investigate whether these associations varied across different populations. This aspect introduced new perspectives for precision medicine and may contribute to the development of targeted health interventions for specific groups. Finally, in the context of the global aging population and the increasing burden of neurodegenerative diseases, our study suggested that dietary interventions may offer a potential avenue for improving brain health [53,54]. Given the modifiable nature of dietary habits, adjusting nutrient intake could play a role in mitigating the risks associated with cognitive decline and promoting healthy aging. Nevertheless, to strengthen these findings and provide more definitive guidance for public health strategies, further experimental studies, such as randomized controlled trials are essential, which would help establish causality and refine dietary recommendations aimed at reducing the burden of neurodegenerative diseases in aging populations.

This study benefited from large sample size and robust data collection methods, allowing for a comprehensive analysis of dietary n-6 PUFA intake and its impact on brain structure in a well-designed cohort. Nonetheless, our study was subject to several limitations. First, the dietary intake data were obtained through a 24-h online questionnaire, which is subject to inherent limitations. The reliance on an online platform may have introduced selection bias, particularly among individuals with limited digital literacy or access [55]. Furthermore, the use of a single 24-h recall is insufficient to represent habitual dietary patterns, potentially leading to measurement error and limiting the accuracy of long-term dietary intake assessment [17]. Second, the identification of brain disorders was based on health records, which may carry inherent constraints [56]. This approach could result in underreporting of cases due to incomplete data capture or reliance on healthcare-seeking behavior, potentially excluding undiagnosed or subclinical cases. Additionally, delays in diagnosis or misclassification within the records may further affect the accuracy of case identification. Third, partial volume effects may have influenced our findings, potentially compromising tissue characterization and estimates of WM microstructure, despite the use of specific imaging techniques designed to mitigate such issues. Fourth, although we accounted for numerous widely accepted confounders, the possibility of residual or unmeasured confounding effects cannot be dismissed. The associations observed through regression modeling do not imply causality, and substantial evidence is needed to support the biological plausibility of our findings. Finally, the study’s sample predominantly consisted of individuals of European descent from the UK, which limits the generalizability of the results to other populations. Meanwhile, volunteer bias was also present, as the sample exhibited a significantly higher rate of never smoking and a lower prevalence of diabetes compared to the general population, potentially leading to an underestimation of certain risk factors and affecting the external validity of the findings. To enhance the evidence base, future research should prioritize minimizing potential biases by employing more precise dietary assessment methods and addressing the inherent limitations of self-reported data. Additionally, more epidemiological studies are needed to validate the associations observed in diverse populations, which would strengthen the applicability of findings across different demographic groups. Furthermore, further investigation is essential to elucidate the mechanisms linking n-6 PUFA intake with brain health through experimental research, including animal studies and randomized controlled trials, which will be pivotal for establishing causal relationships and advancing the development of dietary recommendations.

## 5. Conclusions

In conclusion, our study utilized data from the UK Biobank to investigate the associations of dietary n-6 PUFA intake with neurodegenerative diseases, focusing on DEM, PD, MS, and brain structure alterations. Cox proportional hazard regression and multiple linear regression models were applied to assess these associations, respectively. Our results indicated that lower n-6 PUFA intake was linked to increased risks of DEM, PD, and MS. Neuroimaging results demonstrated that low n-6 PUFA intake was linked to atrophic traits in GM volumes and reduced WM integrity. Our findings underscored the potential importance of dietary n-6 PUFA intake in preserving brain health and mitigating the risk of neurodegenerative diseases. While further research is essential to validate these associations and clarify the underlying mechanisms, our results offered meaningful insights to guide future investigations. Building on this evidence, promoting dietary modifications, particularly among vulnerable populations, may represent a practical strategy to support healthy aging and help alleviate the societal burden of neurodegenerative disorders.

## Figures and Tables

**Table 1:** Baseline characteristics of participants grouped by dietary n-6 PUFA intake level.

Characteristic	All Participants (N = 169,317)	High Intake ^a^ (N = 84,658)	Low Intake (N = 84,659)
Age, year			
<65	101,832 (60.2%)	52,029 (61.5%)	49,803 (58.8%)
≥65	67,463 (39.8%)	32,618 (38.5%)	34,845 (41.2%)
Sex			
Female	89,918 (53.1%)	40,778 (48.2%)	49,140 (58.1%)
Male	79,377 (46.9%)	43,869 (51.8%)	35,508 (41.9%)
IMD ^b^			
Low	84,673 (50.0%)	42,489 (50.2%)	42,184 (49.8%)
High	84,622 (50.0%)	42,158 (49.8%)	42,464 (50.2%)
WHR ^c^			
Ideal	91,990 (54.3%)	44,903 (53.0%)	47,087 (55.6%)
Poor	77,305 (45.7%)	39,744 (47.0%)	37,561 (44.4%)
Healthy Lifestyle			
Never smoking ^d^	155,840 (92.2%)	78,015 (92.3%)	77,825 (92.1%)
No heavy alcohol intake ^e^	111,983 (66.2%)	55,355 (65.4%)	56,628 (66.9%)
Regular physical activity ^f^	91,404 (54.0%)	46,512 (54.9%)	44,892 (53.0%)
Healthy sleep pattern ^g^	95,932 (56.7%)	47,637 (56.3%)	48,295 (57.1%)
Health diet ^h^	69,180 (40.9%)	34,094 (40.3%)	35,086 (41.4%)
Blood pressure			
SBP	135.0 (124.0, 148.0)	134.0 (124.0, 148.0)	135.0 (124.0, 148.0)
DBP	81.5 (74.5, 88.0)	81.5 (74.5, 88.0)	81.5 (75.0, 88.0)
Metabolic biomarkers			
Glucose	5.0 (4.6, 5.3)	4.9 (4.6, 5.3)	5.0 (4.6, 5.3)
HbA1c	34.9 (32.5, 37.4)	34.9 (32.5, 37.4)	34.8 (32.4, 37.4)
TG	1.4 (1.0, 2.1)	1.5 (1.0, 2.1)	1.4 (1.0, 2.0)
LDL	3.5 (3.0, 4.1)	3.5 (2.9, 4.1)	3.5 (3.0, 4.1)
Dietary n-3 PUFA intake	1.81 (1.31, 2.47)	2.26 (1.83, 2.88)	1.36 (1.03, 1.78)
Ratio of dietary n-3/n-6 PUFA intake			
High ^i^	84,647 (50.0%)	33,116 (39.1%)	51,531 (60.9%)
Low	84,648 (50.0%)	51,531 (60.9%)	33,117 (39.1%)

Abbreviations: PUFA, polyunsaturated fatty acids; IMD, indices of multiple deprivation; WHR, waist to hip ratio; SBP, systolic blood pressure; DBP, diastolic blood pressure; HbA1c, glycated hemoglobin; TG, triglycerides; LDL, low-density lipoprotein cholesterol. ^a^ Participants’ intake levels were dichotomized into low-intake and high-intake groups based on the median intake (cut-off value: 10.15 g/d). ^b^ IMD scores offer a more complex and detailed view of deprivation, based on more factors than the Townsend index. Domains for IMD calculation: crime score (England and Scotland), community safety score (Wales), education score (All), employment score (All), health score (All), housing score (All), income score (All), living environment score (England), access to services score (Scotland and Wales), physical environment score (Wales). ^c^ WHR was calculated as waist circumference (centimeter) divided by hip circumference (centimeter) and categorized into ideal (<0.9 for men and <0.85 for women) and poor (≥ 0.9 for men and ≥ 0.85 for women). ^d^ Never smoking was defined for individuals who reported no previous or current smoking at baseline. ^e^ No heavy alcohol intake was defined as the average daily intake ≤16 g of pure alcohol (2 units of alcohol) for both men and women. ^f^ Regular physical activity: ≥150 min of moderate-intensity per week or ≥75 min of vigorous-intensity per week, or a combination of both. ^g^ Healthy sleep pattern was measured by 5 dimensions of sleep behaviors: early chronotype, sleep 7–8 h/day, never/rarely or sometimes insomnia, no self-reported snoring, and never/rarely or sometimes excessive daytime sleepiness, and categorized into yes (≥4 healthy components) and no. ^h^ Healthy diet was characterized by the consumption of at least four out of seven food groups that are commonly recommended as dietary priorities for cardiometabolic health. The specific frequencies for each component of a healthy diet are as follows: ≥3 servings/day for fruit; ≥3 servings/day for vegetables; ≥2 servings/week for fish; ≤1 serving/week processed meats; ≤1.5 servings/week for unprocessed red meats; ≥ 3 servings/day for whole grains; ≤1.5 servings/day for refined grains. ^i^ Participants’ dietary n-3/n-6 PUFA ratios were categorized into low and high levels based on the median value (cut-off value: 0.17).

**Table 2:** Associations between dietary n-6 PUFA intake and neurodegenerative diseases by demographics.

Subgroup	DEM		PD		MS	
	HR	*P* for Interaction ^a^	HR	*P* for Interaction	HR	*P* for Interaction
Age						
40–59	1.14 (0.85, 1.53)	0.379	1.24 (0.86, 1.81)	0.400	1.32 (0.72, 2.42)	0.450
≥60	1.31 (1.13, 1.52)		1.48 (1.19, 1.84)		1.82 (0.90, 3.69)	
60–64	1.72 (1.38, 2.14)	<0.001	1.49 (1.09, 2.05)	0.774	3.11 (1.10, 8.84)	0.126
≥65	1.08 (0.91, 1.29)		1.41 (1.08, 1.85)		1.10 (0.44, 2.77)	
Sex						
Male	1.31 (1.11, 1.54)	0.854	1.36 (1.08, 1.71)	0.434	1.77 (0.89, 3.53)	0.798
Female	1.28 (1.05, 1.56)		1.57 (1.14, 2.17)		1.60 (0.92, 2.76)	
IMD ^b^						
Low	1.39 (1.16, 1.66)	0.283	1.50 (1.16, 1.94)	0.491	1.37 (0.75, 2.49)	0.317
High	1.23 (1.03, 1.46)		1.34 (1.03, 1.74)		2.01 (1.09, 3.70)	
WHR ^c^						
Poor	1.35 (1.15, 1.59)	0.393	1.36 (1.07, 1.74)	0.552	1.40 (0.76, 2.59)	0.416
Ideal	1.22 (1.01, 1.49)		1.51 (1.14, 2.00)		1.92 (1.06, 3.47)	

Abbreviations: PUFA, polyunsaturated fatty acids; DEM, dementia; PD, Parkinson’s disease; MS, multiple sclerosis; HR, hazard ratio; IMD, indices of multiple deprivation; WHR, waist to hip ratio. ^a^ A low ‘*P* for interaction’ (typically less than 0.05) suggests statistical significance. ^b^ IMD scores offer a more complex and detailed view of deprivation, based on more factors than the Townsend index. Domains for IMD calculation: crime score (England and Scotland), community safety score (Wales), education score (All), employment score (All), health score (All), housing score (All), income score (All), living environment score (England), access to services score (Scotland and Wales), physical environment score (Wales). ^c^ WHR was calculated as waist circumference (centimeter) divided by hip circumference (centimeter) and categorized into ideal (<0.9 for men and <0.85 for women) and poor (≥0.9 for men and ≥0.85 for women).

**Table 3:** Associations between dietary n-6 PUFA intake and neurodegenerative diseases by prevalent diseases, physical frailty, and telomere length.

Subgroup	DEM		PD		MS	
	HR	*P* for Interaction ^a^	HR	*P* for Interaction	HR	*P* for Interaction
Hypertension						
Yes	1.35 (1.12, 1.62)	0.516	1.47 (1.10, 1.97)	0.721	1.97 (0.80, 4.84)	0.654
No	1.25 (1.05, 1.48)		1.39 (1.09, 1.76)		1.59 (0.96, 2.64)	
Diabetes						
Yes	1.32 (0.93, 1.87)	0.914	1.45 (0.86, 2.46)	0.917	1.03 (0.14, 7.51)	0.632
No	1.29 (1.12, 1.49)		1.41 (1.15, 1.74)		1.68 (1.04, 2.71)	
Physical frailty ^b^						
Yes	1.31 (1.08, 1.57)	0.452	1.37 (1.04, 1.82)	0.914	1.78 (0.96, 3.30)	0.577
No	1.20 (1.00, 1.43)		1.40 (1.09, 1.79)		1.44 (0.79, 2.61)	
Telomere length ^c^						
Short	1.32 (1.12, 1.57)	0.358	1.43 (1.11, 1.84)	0.566	1.99 (0.90, 4.38)	0.814
Long	1.19 (0.98, 1.45)		1.29 (0.98, 1.72)		1.79 (1.03, 3.11)	

Abbreviations: PUFA, polyunsaturated fatty acids; DEM, dementia; PD, Parkinson’s disease; MS, multiple sclerosis; HR, hazard ratio. ^a^ A low ‘*P* for interaction’ (typically less than 0.05) suggests statistical significance. ^b^ Physical frailty was defined using multiple criteria, often based on markers that reflect muscle strength, physical performance, and self-reported limitations in daily activities. ^c^ Telomere length was measured using biological samples, particularly blood leukocyte DNA, in a subset of UK Biobank participants. It is an indicator of biological aging. Shorter telomere length is associated with increased aging and susceptibility to diseases.

## Data Availability

All the data for this study will be made available upon reasonable request to the corresponding authors.

## Supplementary Materials

The following supporting information can be downloaded at: https://www.mdpi.com/article/10.3390/nu16244272/s1, Table S1: definitions of neurodegenerative diseases; Table S2: definitions of imaging phenotypes of brain gray matter; Table S3: definitions of covariates; Table S4: single-nucleotide polymorphisms used to build the genetic risk score for DEM; Table S5: single-nucleotide polymorphisms used to build the genetic risk score for PD; Table S6: single-nucleotide polymorphisms used to build the genetic risk score for MS; Table S7: incidence rates of neurodegenerative diseases by sex; Table S8: associations of dietary n-3 PUFA intake and neurodegenerative diseases; Table S9: descriptive analysis of brain phenotypes; Table S10: associations of dietary n-6 PUFA intake with phenotypes of gray matter by sex; Table S11: associations of dietary n-6 PUFA intake with phenotypes of white matter by sex; Table S12: associations of dietary n-6 PUFA intake with the phenotypes of brain gray matter; Table S13: associations of dietary n-6 PUFA intake with the phenotypes of brain white matter; Table S14: sensitivity analysis of the main associations.
