# Supplementary material for: Dietary N-6 Polyunsaturated Fatty Acid Intake and Brain Health in Middle-Aged and Elderly Adults
Source: Nutrients. 2024 Dec 11;16(24):4272. doi: 10.3390/nu16244272 (PMC11680004; doi:10.3390/nu16244272)
Supplement: Supplementary file 1 [file nutrients-16-04272-s001.zip › nutrients-3309787-supplementary.pdf]

Supplementary Information

Dietary N-6 Polyunsaturated Fatty Acids Intake and Brain Health in Middle-Aged and Elderly Adults

Table S1. Definitions of neurodegenerative diseases ... 2

Table S2. Definitions of imaging phenotypes of brain gray matter and white matter ... 3

Table S3. Definitions of covariates ... 6

Table S4. Single-nucleotide polymorphisms used to build the genetic risk score for DEM ... 7

Table S5. Single-nucleotide polymorphisms used to build the genetic risk score for PD ... 9

Table S6. Single-nucleotide polymorphisms used to build the genetic risk score for MS ... 10

Table S7. Incidence rates of neurodegenerative diseases by sex ... 13

Table S8. Associations of dietary n-3 PUFA intake and neurodegenerative diseases ... 14

Table S9. Descriptive analysis of brain phenotypes ... 15

Table S10. Associations of dietary n-6 PUFA intake with phenotypes of gray matter by sex ... 18

Table S11. Associations of dietary n-6 PUFA intake with phenotypes of white matter by sex ... 20

Table S13. Associations of dietary n-6 PUFA intake with the phenotypes of brain white matter ... 22

Table S14. Sensitivity analysis of the main associations ... 23

Table S1. Definitions of neurodegenerative diseases

Neurodegenerative diseases	Abbr	Source
Dementia	DEM	**New case identification:**<br>ICD-9: 290, 291.2, 294.1, 331<br>ICD-10: F00, F01, F02, F03, F05.1, F10.6, G30, G31, I67.3
Parkinson's disease	PD	**New case identification:**<br>ICD-9: 332<br>ICD-10: G20
Multiple sclerosis	MS	**New case identification:**<br>ICD-9: 340<br>ICD-10: G35

Table S2. Definitions of imaging phenotypes of brain gray matter and white matter

Entity	Abbr	Unit	Source
**Volume of subcortical structure**			
accumbens nucleus	NAc	mm^3	UKB data field 25023 and 25024
amygdala	AMYG	mm^3	UKB data field 25021 and 25022
caudate nucleus	CN	mm^3	UKB data field 25013 and 25014
hippocampus	HIP	mm^3	UKB data field 25019 and 25020
globus pallidus	GP	mm^3	UKB data field 25017 and 25018
putamen	PUT	mm^3	UKB data field 25015 and 25016
thalamus	THA	mm^3	UKB data field 25011 and 25012
**Volume of cortical structure**			
**Frontal lobe**			
caudal anterior cingulate cortex	cACC	mm^3	UKB data field 26790 and 26891
caudal middle frontal gyrus	cMFG	mm^3	UKB data field 26791 and 26892
frontal pole	FP	mm^3	UKB data field 26819 and 26920
lateral orbitofrontal cortex	lOFC	mm^3	UKB data field 26799 and 26900
medial orbitofrontal cortex	mOFC	mm^3	UKB data field 26801 and 26902
paracentral lobule	PCL	mm^3	UKB data field 26804 and 26905
pars opercularis	Op	mm^3	UKB data field 26805 and 26906
pars orbitalis	Or	mm^3	UKB data field 26806 and 26907
pars triangularis	Tr	mm^3	UKB data field 26807 and 26908
precentral gyrus	PCG	mm^3	UKB data field 26811 and 26912
rostral anterior cingulate cortex	rACC	mm^3	UKB data field 26813 and 26914
rostral middle frontal gyrus	RMFG	mm^3	UKB data field 26814 and 26915
superior frontal gyrus	SFG	mm^3	UKB data field 26815 and 26916
**Temporal lobe**			
banks of superior temporal sulcus	BANKSSTS	mm^3	UKB data field 26789 and 26890
entorhinal cortex	EC	mm^3	UKB data field 26793 and 26894
fusiform gyrus	FG	mm^3	UKB data field 26794 and 26895
inferior temporal gyrus	ITG	mm^3	UKB data field 26796 and 26897
middle temporal gyrus	MTG	mm^3	UKB data field 26802 and 26903
parahippocampal gyrus	PHG	mm^3	UKB data field 26803 and 26904
superior temporal gyrus	STG	mm^3	UKB data field 26817 and 26918
transverse temporal gyrus	TTG	mm^3	UKB data field 26820 and 26921
**Parietal lobe**			
inferior parietal lobule	IPL	mm^3	UKB data field 26795 and 26896
isthmus cingulate	Isthmus	mm^3	UKB data field 26797 and 26898
postcentral gyrus	PoC	mm^3	UKB data field 26809 and 26910
posterior cingulate cortex	PCC	mm^3	UKB data field 26810 and 26911
precuneus	PCUN	mm^3	UKB data field 26812 and 26913
superior parietal lobule	SPL	mm^3	UKB data field 26816 and 26917
supramarginal gyrus	SMG	mm^3	UKB data field 26818 and 26919
**Occipital lobe**			

cuneus	CUN	mm^3	UKB data field 26792 and 26893
lateral occipital cortex	LOC	mm^3	UKB data field 26798 and 26899
lingual gyrus	LG	mm^3	UKB data field 26800 and 26901
pericalcarine cortex	PCAL	mm^3	UKB data field 26808 and 26909
**Insula lobe**			
insula	INS	mm^3	UKB data field 26821 and 26922
**White matter tracts**			
**FA values**			
tract forceps major	FMA	AU	UKB data field 25498
tract forceps minor	FMI	AU	UKB data field 25499
tract middle cerebellar peduncle	MCP	AU	UKB data field 25504
tract acoustic radiation	AR	AU	UKB data field 25488 and 25489
tract anterior thalamic radiation	ATR	AU	UKB data field 25490 and 25491
tract cingulate gyrus part of cingulum	CGC	AU	UKB data field 25492 and 25493
tract corticospinal tract	CST	AU	UKB data field 25496 and 25497
tract inferior fronto-occipital fasciculus	IFOF	AU	UKB data field 25500 and 25501
tract inferior longitudinal fasciculus	ILF	AU	UKB data field 25502 and 25503
tract medial lemniscus	ML	AU	UKB data field 25505 and 25506
tract parahippocampal part of cingulum	CGH	AU	UKB data field 25494 and 25495
tract posterior thalamic radiation	PTR	AU	UKB data field 25507 and 25508
tract superior longitudinal fasciculus	SLF	AU	UKB data field 25509 and 25510
tract superior thalamic radiation	STR	AU	UKB data field 25511 and 25512
tract uncinate fasciculus	UF	AU	UKB data field 25513 and 25514
**MD values**			
tract forceps major	FMA	AU	UKB data field 25525
tract forceps minor	FMI	AU	UKB data field 25526
tract middle cerebellar peduncle	MCP	AU	UKB data field 25531
tract acoustic radiation	AR	AU	UKB data field 25515 and 25516
tract anterior thalamic radiation	ATR	AU	UKB data field 25517 and 25518
tract cingulate gyrus part of cingulum	CGC	AU	UKB data field 25519 and 25520
tract corticospinal tract	CST	AU	UKB data field 25523 and 25524
tract inferior fronto-occipital fasciculus	IFOF	AU	UKB data field 25527 and 25528
tract inferior longitudinal fasciculus	ILF	AU	UKB data field 25529 and 25530
tract medial lemniscus	ML	AU	UKB data field 25532 and 25533
tract parahippocampal part of cingulum	CGH	AU	UKB data field 25521 and 25522
tract posterior thalamic radiation	PTR	AU	UKB data field 25534 and 25535
tract superior longitudinal fasciculus	SLF	AU	UKB data field 25536 and 25537
tract superior thalamic radiation	STR	AU	UKB data field 25538 and 25539
tract uncinate fasciculus	UF	AU	UKB data field 25540 and 25541
**ICVF values**			
tract forceps major	FMA	AU	UKB data field 25660
tract forceps minor	FMI	AU	UKB data field 25661
tract middle cerebellar peduncle	MCP	AU	UKB data field 25666

tract acoustic radiation	AR	AU	UKB data field 25650 and 25651
tract anterior thalamic radiation	ATR	AU	UKB data field 25652 and 25653
tract cingulate gyrus part of cingulum	CGC	AU	UKB data field 25654 and 25655
tract corticospinal tract	CST	AU	UKB data field 25658 and 25659
tract inferior fronto-occipital fasciculus	IFOF	AU	UKB data field 25662 and 25663
tract inferior longitudinal fasciculus	ILF	AU	UKB data field 25664 and 25665
tract medial lemniscus	ML	AU	UKB data field 25667 and 25668
tract parahippocampal part of cingulum	CGH	AU	UKB data field 25656 and 25657
tract posterior thalamic radiation	PTR	AU	UKB data field 25669 and 25670
tract superior longitudinal fasciculus	SLF	AU	UKB data field 25671 and 25672
tract superior thalamic radiation	STR	AU	UKB data field 25673 and 25674
tract uncinate fasciculus	UF	AU	UKB data field 25675 and 25676

Table S3. Definitions of covariates

**Entity**	**Abbr**	**Unit**	**Source**
Age		year	UKB data field 21022
Sex		N/A	UKB data field 31
Index of multiple deprivation	IMD	N/A	UKB data field 26410, 26427, 26426
Waist-hip rate	WHR	N/A	UKB data field 48, 49
Never smoking		N/A	UKB data field 20116
No heavy alcohol intake		N/A	UKB data field 20117, 1558, 1568, 1578, 1588, 1598, 1608, 4407, 4418, 4429, 4440, 4451
Healthy sleep pattern		N/A	UKB data field 1180, 1160, 1200, 1210, 1220
Regular physical activity		N/A	UKB data field 22035
Healthy diet		N/A	UKB data field 1309, 1319, 1289, 1299, 1329, 1339, 1349, 1369, 1379, 1389, 1438, 1448, 1458, 1468
Systolic blood pressure	SBP	mmHg	UKB data field 4080, 93
Diastolic blood pressure	DBP	mmHg	UKB data field 4079, 94
Glucose	Glc	mmol/L	UKB data field 30740
Glycated hemoglobin	HbA1c	mmol/mol	UKB data field 30750
Triglycerides	TG	mmol/L	UKB data field 30870
Low-density lipoprotein	LDL	mmol/L	UKB data field 30780
Physical frailty	PF	N/A	UKB data field 2306, 2080, 31, 21022, 22037, 22038, 22039, 924, 21001, 46, 47

Table S4. Single-nucleotide polymorphisms used to build the genetic risk score for DEM

SNP rsID	Chr	Position	Effect Allele	Beta	*P* value
rs679515	1	207577223	T	0.122217633	7.2×10^{-46}
rs141749679	1	109345810	C	0.322083499	7.5×10^{-9}
rs6733839	2	127135234	T	0.157003749	6.1×10^{-118}
rs10933431	2	233117202	G	-0.072570693	3.6×10^{-18}
rs72777026	2	9558882	G	0.058268908	2.7×10^{-8}
rs17020490	2	37304796	C	0.058268908	3.3×10^{-9}
rs143080277	2	105749599	C	0.385262401	2.1×10^{-13}
rs139643391	2	202878716	T	-0.061875404	1.1×10^{-8}
rs16824536	3	155069722	A	-0.083381609	3.6×10^{-8}
rs61762319	3	155084189	G	0.148420005	2.2×10^{-11}
rs6846529	4	11023507	C	0.067658648	2.2×10^{-17}
rs3822030	4	993555	G	-0.051293294	8.3×10^{-12}
rs2245466	4	40197226	G	0.048790164	1.2×10^{-9}
rs112403360	5	14724304	A	0.086177696	2.3×10^{-9}
rs62374257	5	86927378	C	0.067658648	1.4×10^{-15}
rs871269	5	151052827	T	-0.040821995	8.7×10^{-9}
rs113706587	5	180201150	A	0.086177696	2.2×10^{-16}
rs6605556	6	32615322	G	-0.094310679	7.1×10^{-20}
rs10947943	6	41036354	A	-0.061875404	1.1×10^{-9}
rs143332484	6	41161469	T	0.343589704	2.8×10^{-25}
rs75932628	6	41161514	T	0.871293366	2.5×10^{-37}
rs60755019	6	41181270	G	0.438254931	2.1×10^{-8}
rs7767350	6	47517390	T	0.076961041	7.9×10^{-22}
rs785129	6	114291731	T	0.039220713	2.4×10^{-9}
rs6966331	7	37844191	T	-0.040821995	4.6×10^{-10}
rs7384878	7	100334426	C	-0.083381609	1.1×10^{-26}
rs11771145	7	143413669	A	-0.051293294	3.3×10^{-14}
rs6943429	7	7817263	T	0.048790164	1.0×10^{-10}
rs10952097	7	8204382	T	0.067658648	6.8×10^{-9}
rs13237518	7	12229967	A	-0.040821995	4.9×10^{-11}
rs1160871	7	28129126	G	-0.051293294	9.8×10^{-9}
rs76928645	7	54873635	T	-0.072570693	1.6×10^{-10}
rs73223431	8	27362470	T	0.067658648	4.0×10^{-22}
rs11787077	8	27607795	T	-0.094310679	1.7×10^{-44}
rs1065712	8	11844613	C	0.086177696	1.9×10^{-9}
rs34173062	8	144103704	A	0.122217633	1.7×10^{-16}
rs1800978	9	104903697	G	0.058268908	1.6×10^{-9}
rs7912495	10	11676714	G	0.058268908	9.7×10^{-19}
rs7068231	10	60025170	T	-0.051293294	3.3×10^{-13}
rs6586028	10	80494228	C	-0.072570693	2.0×10^{-19}
rs6584063	10	96266650	G	-0.116533816	6.7×10^{-11}
rs7908662	10	122413396	G	-0.040821995	2.6×10^{-9}
rs10437655	11	47370397	A	0.058268908	5.3×10^{-14}
rs1582763	11	60254475	A	-0.094310679	3.7×10^{-42}
rs3851179	11	86157598	T	-0.105360516	3.0×10^{-48}
rs74685827	11	121482368	G	0.173953307	2.8×10^{-11}
rs11218343	11	121564878	C	-0.174353387	1.4×10^{-21}

rs6489896	12	113281983	C	0.076961041	1.8×10^{-9}
rs17125924	14	52924962	G	0.09531018	8.3×10^{-16}
rs7401792	14	92464917	G	0.039220713	4.8×10^{-8}
rs12590654	14	92472511	A	-0.072570693	4.2×10^{-21}
rs7157106	14	105761758	A	0.048790164	2.0×10^{-8}
rs10131280	14	106665591	A	-0.061875404	4.3×10^{-10}
rs8025980	15	50701814	G	-0.040821995	1.3×10^{-8}
rs602602	15	58764824	A	-0.061875404	2.1×10^{-15}
rs117618017	15	63277703	T	0.104360015	2.2×10^{-25}
rs3848143	15	64131307	G	0.048790164	8.4×10^{-11}
rs12592898	15	78936857	A	-0.061875404	4.2×10^{-9}
rs889555	16	31111250	T	-0.051293294	2.0×10^{-11}
rs4985556	16	70660097	A	0.067658648	6.0×10^{-10}
rs12446759	16	81739398	G	-0.051293294	1.2×10^{-13}
rs72824905	16	81908423	G	-0.301105093	8.5×10^{-12}
rs1140239	16	30010081	T	-0.061875404	2.6×10^{-13}
rs450674	16	79574511	C	-0.040821995	3.2×10^{-8}
rs16941239	16	86420604	A	0.122217633	1.3×10^{-8}
rs56407236	16	90103687	A	0.104360015	6.5×10^{-15}
rs7225151	17	5233752	A	0.076961041	4.1×10^{-13}
rs199515	17	46779275	G	-0.061875404	9.3×10^{-13}
rs616338	17	49219935	T	0.277631737	2.8×10^{-14}
rs2526377	17	58332680	G	-0.051293294	1.6×10^{-12}
rs4277405	17	63471557	C	-0.061875404	8.8×10^{-20}
rs35048651	17	1728046	T	0.058268908	7.7×10^{-11}
rs2242595	17	18156140	A	-0.061875404	1.1×10^{-9}
rs5848	17	44352876	T	0.067658648	2.4×10^{-20}
rs12151021	19	1050875	A	0.09531018	1.6×10^{-37}
rs149080927	19	1854254	G	0.048790164	5.1×10^{-10}
rs9304690	19	49950060	T	0.048790164	4.7×10^{-9}
rs587709	19	54267597	C	0.048790164	3.6×10^{-11}
rs6014724	20	56423488	G	-0.116533816	4.1×10^{-21}
rs1358782	20	413334	A	-0.051293294	1.6×10^{-8}
rs6742	20	63743088	T	-0.051293294	2.6×10^{-9}
rs2830489	21	26775872	T	-0.051293294	1.7×10^{-10}
rs2154481	21	26101558	C	-0.051293294	1.0×10^{-12}

Table S5. Single-nucleotide polymorphisms used to build the genetic risk score for PD

SNP rsID	Chr	Position	Effect Allele	Beta	*P* value
rs10797576	1	232664611	T	0.122217633	1.76×10^{-10}
rs10906923	10	15569598	C	-0.072570693	2.37×10^{-8}
rs11060180	12	123303586	G	-0.094310679	3.08×10^{-11}
rs11158026	14	55348869	T	-0.094310679	2.88×10^{-10}
rs11343	16	19279464	T	0.067658648	1.46×10^{-9}
rs115185635	3	87520857	C	0.58221562	2.2×10^{-8}
rs11724635	4	15737101	C	-0.116533816	4.26×10^{-17}
rs117896735	10	121536327	A	0.570979547	1.21×10^{-11}
rs12456492	18	40673380	G	0.09531018	2.15×10^{-11}
rs12497850	3	48748989	G	-0.072570693	6.8×10^{-8}
rs12637471	3	182762437	A	-0.174353387	5.38×10^{-22}
rs13294100	9	17579690	T	-0.094310679	1.99×10^{-12}
rs14235	16	31121793	A	0.09531018	3.63×10^{-12}
rs143918452	3	52816840	G	-0.385662481	2.25×10^{-7}
rs1474055	2	169110394	C	-0.198450939	7.11×10^{-16}
rs1555399	14	67984370	T	0.139761942	5.7×10^{-16}
rs17649553	17	43994648	T	-0.261364764	6.11×10^{-49}
rs199347	7	23293746	G	-0.105360516	5.62×10^{-14}
rs2280104	8	22525980	T	0.058268908	9.06×10^{-7}
rs2414739	15	61994134	G	-0.105360516	3.59×10^{-12}
rs2694528	5	60273923	C	0.139761942	1.69×10^{-11}
rs2740594	8	11707174	A	0.09531018	9.54×10^{-11}
rs329648	11	133765367	T	0.104360015	8.05×10^{-12}
rs34043159	2	102413116	C	0.067658648	3.83×10^{-8}
rs34311866	4	951947	C	0.231111721	6×10^{-41}
rs353116	2	166133632	T	-0.061875404	9.73×10^{-7}
rs356182	4	90626111	G	0.292669614	1.85×10^{-82}
rs35749011	1	155135036	G	-0.562118918	6.1×10^{-23}
rs3793947	11	83544472	A	-0.094310679	2.59×10^{-8}
rs4073221	3	18277488	G	0.104360015	3.02×10^{-9}
rs4653767	1	226916078	C	-0.083381609	2.4×10^{-10}
rs4784227	16	52599188	T	0.076961041	8.29×10^{-8}
rs591323	8	16697091	A	-0.094310679	3.17×10^{-8}
rs601999	17	40698158	C	-0.072570693	8.03×10^{-9}
rs62120679	19	2363319	T	0.131028262	2.52×10^{-9}
rs6430538	2	135539967	T	-0.127833372	3.35×10^{-19}
rs6812193	4	77198986	T	-0.094310679	1.85×10^{-11}
rs76904798	12	40614434	T	0.148420005	4.86×10^{-14}
rs78738012	4	114360372	C	0.131028262	2.11×10^{-9}
rs8005172	14	88472612	T	0.076961041	1.2×10^{-9}
rs8118008	20	3168166	A	0.104360015	2.32×10^{-8}
rs823118	1	205723573	C	-0.116533816	1.96×10^{-16}
rs9275326	6	32666660	T	-0.223143551	5.81×10^{-13}
rs9468199	6	27681215	A	0.113328685	3.44×10^{-13}

Table S6. Single-nucleotide polymorphisms used to build the genetic risk score for MS

SNP rsID	Chr	Position	Effect Allele	Beta	*P* value
rs3748817	1	2525665	A	0.131028262	1.331E-12
rs3007421	1	6530189	A	0.113328685	9.611E-07
rs12087340	1	85746993	A	0.198850859	5.133E-12
rs11587876	1	85915183	A	0.113328685	8.395E-08
rs41286801	1	92975464	A	0.182321557	7.921E-16
rs7552544	1	101240893	A	0.076961041	0.000003674
rs11581062	1	101407519	G	0.048790164	0.012
rs6677309	1	117080166	A	0.292669614	1.454E-28
rs666930	1	120258970	G	0.086177696	7.493E-08
rs2050568	1	157770241	G	0.076961041	0.000001326
rs35967351	1	160711804	A	0.086177696	0.000001702
rs1359062	1	192541472	C	0.165514438	1.842E-13
rs55838263	1	200874728	A	0.113328685	1.408E-09
rs4665719	2	25017860	G	0.086177696	0.000006803
rs2163226	2	43361256	A	0.09531018	7.021E-08
rs842639	2	61095245	A	0.104360015	1.7E-09
rs7595717	2	68587477	A	0.09531018	3.292E-07
rs17174870	2	112665201	G	0.029558802	0.08835
rs9967792	2	191974435	G	0.104360015	1.799E-09
rs9989735	2	231115454	C	0.157003749	7.841E-14
rs11719975	3	18785585	C	0.086177696	0.000005392
rs2371108	3	27757018	A	0.076961041	0.000002055
rs1813375	3	28078571	A	0.139761942	5.748E-18
rs4679081	3	33013483	G	0.076961041	0.00001204
rs9828629	3	71530346	G	0.076961041	0.000005489
rs2028597	3	105558837	G	0.039220713	0.1786
rs1131265	3	119222456	C	0.173953307	1.973E-15
rs1920296	3	121543577	C	0.131028262	6.75E-15
rs2255214	3	121770539	C	0.104360015	1.72E-10
rs9282641	3	121796768	G	0.113328685	0.000586
rs1014486	3	159691112	G	0.104360015	1.159E-09
rs7665090	4	103551603	G	0.076961041	0.000002412
rs2726518	4	106173199	C	0.086177696	0.00001225
rs6881706	5	35879156	C	0.113328685	4.867E-09
rs6880778	5	40399096	G	0.09531018	1.698E-08
rs71624119	5	55440730	G	0.113328685	2.696E-09
rs756699	5	133446575	A	0.113328685	0.000002972
rs371522651	5	141506564	C	0.067658648	0.00005955
rs2546890	5	158759900	A	0.058268908	0.0006591
rs4976646	5	176788570	G	0.122217633	1.042E-12
rs17119	6	14719496	A	0.104360015	0.000001905
rs941816	6	36375304	G	0.122217633	4.468E-09
rs72928038	6	90976768	A	0.104360015	7.626E-07
rs802734	6	128278798	A	0.029558802	0.1577
rs11154801	6	135739355	A	0.104360015	2.345E-09
rs17066096	6	137452908	G	0.131028262	5.906E-12
rs7769192	6	137962655	G	0.076961041	0.000013

rs67297943	6	138244816	A	0.113328685	4.831E-08
rs212405	6	159470559	T	0.139761942	1.428E-15
rs1843938	7	3113034	A	0.076961041	0.000002209
rs706015	7	27014988	C	0.131028262	1.294E-09
rs917116	7	28172739	C	0.113328685	2.065E-08
rs60600003	7	37382465	C	0.148420005	2.533E-08
rs201847125	7	50325567	G	0.104360015	2.913E-08
rs354033	7	149289464	G	0.029558802	0.07696
rs1021156	8	79575804	A	0.113328685	5.595E-10
rs2456449	8	128192981	G	0.09531018	2.21E-08
rs4410871	8	128815029	G	0.113328685	1.983E-09
rs759648	8	129158945	C	0.086177696	0.000002818
rs2150702	9	5893861	G	0.148420005	0.000000033
rs2104286	10	6099045	A	0.19062036	7.608E-23
rs793108	10	31415106	A	0.086177696	5.608E-08
rs2688608	10	75658349	A	0.067658648	0.00006372
rs1782645	10	81048611	A	0.086177696	0.00000043
rs7923837	10	94481917	G	0.104360015	4.575E-09
rs7120737	11	47702395	G	0.122217633	7.606E-08
rs34383631	11	60793330	A	0.104360015	5.689E-10
rs694739	11	64097233	A	0.076961041	0.00001303
rs533646	11	118566746	G	0.09531018	0.00000036
rs9736016	11	118724894	T	0.09531018	0.000000022
rs523604	11	118755738	A	0.086177696	0.00000025
rs1800693	12	6440009	G	0.131028262	6.922E-16
rs12296430	12	6503500	C	0.131028262	3.618E-10
rs11052877	12	9905690	G	0.09531018	5.369E-09
rs201202118	12	58182062	A	0.131028262	7.403E-13
rs7132277	12	123593382	A	0.09531018	0.000001876
rs4772201	13	100086259	A	0.113328685	1.672E-07
rs2236262	14	69261472	A	0.076961041	0.00001157
rs4903324	14	75961511	A	0.09531018	0.000009615
rs74796499	14	88432328	C	0.270027137	8.474E-11
rs12148050	14	103263788	A	0.076961041	0.00001467
rs59772922	15	79207466	A	0.104360015	0.000004016
rs8042861	15	90977333	A	0.076961041	9.801E-07
rs2744148	16	1073552	G	0.086177696	0.0001016
rs12927355	16	11194771	G	0.19062036	8.188E-27
rs4780346	16	11288806	A	0.086177696	0.0000068
rs6498184	16	11435990	G	0.139761942	2.067E-10
rs7204270	16	30156963	G	0.086177696	9.324E-08
rs1886700	16	68685905	A	0.104360015	0.000008755
rs12149527	16	79110596	A	0.076961041	0.000001738
rs7196953	16	79649394	A	0.076961041	0.00002645
rs35929052	16	85994484	G	0.131028262	3.316E-07
rs12946510	17	37912377	A	0.076961041	0.000008512
rs4796791	17	40530763	A	0.09531018	1.809E-08
rs4794058	17	45597098	A	0.067658648	0.00001633
rs8070345	17	57816757	A	0.131028262	5.434E-16

rs7238078	18	56384192	A	0.048790164	0.006288
rs1077667	19	6668972	G	0.148420005	3.536E-13
rs34536443	19	10463118	C	0.246860078	1.244E-08
rs2288904	19	10742170	G	0.131028262	9.57E-10
rs1870071	19	16505106	G	0.113328685	5.678E-10
rs11554159	19	18285944	G	0.139761942	2.581E-13
rs8107548	19	49870643	G	0.086177696	0.000001976
rs4810485	20	44747947	A	0.076961041	0.00001783
rs17785991	20	48438761	A	0.086177696	6.421E-07
rs2248359	20	52791518	G	0.067658648	0.00009811
rs2256814	20	62373983	A	0.104360015	8.341E-07
rs6062314	20	62409713	A	0.09531018	0.003871
rs2283792	22	22131125	C	0.076961041	0.000001137
rs470119	22	50966914	A	0.067658648	0.0001508

Table S7. Incidence rates of neurodegenerative diseases by sex

Disease outcome	New events, No	Total person years, year	Incidence Rate, per 1,000 person-years
**All**			
DEM	1534	2255560	68.0
PD	770	2257284	34.1
MS	159	2259315	7.0
**Male**			
DEM	918	1050385	87.4
PD	520	1051086	49.5
MS	51	1052776	4.8
**Female**			
DEM	616	1205175	51.1
PD	250	1206197	20.7
MS	108	1206538	9.0

Abbreviations: DEM, dementia; PD, Parkinson’s disease; MS, multiple sclerosis.

Table S8. Associations of dietary n-3 PUFA intake and neurodegenerative diseases.

Characteristic	DEM		PD		MS	
	HR	*P* value[a]	HR	*P* value	HR	*P* value
**High intake**[b]	Reference	0.003	Reference	0.033	Reference	0.310
**Low intake**	1.27 (1.09, 1.49)		1.29 (1.02, 1.63)		1.33 (0.77, 2.31)	

Abbreviations: PUFA, polyunsaturated fatty acids; DEM, dementia; PD, Parkinson's disease; MS, multiple sclerosis; HR, hazard ratio. **a** A low 'P value ' (typically less than 0.05) suggests statistical significance. **b** Dietary n-3 PUFA intake was categorized into low and high levels based on their median values in the study population (cutoff value: 1.81 g/d).

Table S9. Descriptive analysis of brain phenotypes

Phenotype[a]	P25	P50	P75	Mean	SD
**Volume of subcortical structure**					
NAc	371.500	441.000	512.000	441.911	105.871
AMYG	1,101.500	1,248.000	1,390.000	1,249.202	215.007
CN	3,190.000	3,457.500	3,740.625	3,474.731	414.343
HIP	3,567.000	3,852.250	4,130.000	3,841.716	440.870
GP	1,629.500	1,762.000	1,915.500	1,783.367	237.638
PUT	4,422.500	4,786.750	5,170.125	4,806.041	571.159
THA	7,156.500	7,641.500	8,144.000	7,667.432	732.250
**Volume of cortex**					
**Frontal lobe**					
cACC	1,606.000	1,862.500	2,124.750	1,880.042	401.281
cMFG	5,868.375	6,488.750	7,166.625	6,555.582	984.318
FP	988.000	1,069.500	1,160.500	1,079.085	134.399
lOFC	7,513.875	8,075.000	8,640.625	8,104.614	841.230
mOFC	5,606.875	6,011.750	6,458.000	6,046.120	638.665
PCL	3,680.875	3,991.500	4,332.625	4,027.701	494.655
Op	4,107.250	4,472.750	4,881.750	4,517.949	594.467
Or	2,465.500	2,674.000	2,895.125	2,690.119	325.560
Tr	3,729.875	4,076.500	4,476.625	4,125.319	563.084
PCG	13,078.125	14,061.000	15,206.500	14,158.624	1,598.618
rACC	2,243.375	2,520.000	2,826.000	2,545.329	430.002
RMFG	14,763.375	16,097.500	17,619.125	16,280.085	2,146.356
SFG	21,329.875	23,135.500	25,006.375	23,286.289	2,727.892
**Temporal lobe**					
BANKSSTS	2,195.250	2,413.750	2,657.500	2,438.134	348.451
EC	1,676.500	1,885.750	2,107.500	1,909.049	339.937
FG	9,424.375	10,227.500	11,072.000	10,295.935	1,232.887
ITG	10,646.750	11,622.500	12,697.375	11,718.371	1,529.882
MTG	10,892.875	11,858.250	12,903.125	11,938.877	1,497.219
PHG	1,813.500	1,973.250	2,147.500	1,984.882	255.463
STG	11,613.250	12,547.750	13,538.625	12,627.444	1,450.065
TTG	1,004.000	1,116.000	1,246.000	1,132.270	180.684
**Parietal lobe**					
IPL	12,633.000	13,754.500	15,084.250	13,893.863	1,818.206
Isthmus	2,411.000	2,659.000	2,924.000	2,685.844	387.712
PoC	9,273.875	10,037.750	10,898.500	10,127.873	1,250.854
PCC	3,103.000	3,391.500	3,698.000	3,409.899	452.339
PCUN	9,709.250	10,497.500	11,369.625	10,578.518	1,266.924
SPL	12,934.875	14,001.250	15,132.125	14,058.776	1,644.182
SMG	10,404.375	11,407.500	12,496.125	11,513.895	1,567.085
**Occipital lobe**					
CUN	2,820.500	3,139.750	3,516.500	3,195.762	527.076

LOC	11,525.500	12,605.500	13,770.875	12,706.513	1,667.650
LG	5,915.875	6,588.000	7,349.500	6,651.593	1,055.347
PCAL	1,949.500	2,219.500	2,534.000	2,261.000	434.152
**Insular lobe**					
INS	6,834.000	7,337.250	7,907.125	7,393.928	794.152
**White matter tracts**					
**FA values**					
FMA	0.565399	0.583739	0.599285	0.580973	0.027348
FMI	0.449267	0.463334	0.476595	0.462461	0.020749
MCP	0.463652	0.481357	0.498531	0.480625	0.028082
AR	0.404043	0.416842	0.429037	0.416857	0.019091
ATR	0.382782	0.394676	0.405571	0.393578	0.017794
CGC	0.493211	0.513507	0.532324	0.512693	0.029606
CST	0.526567	0.541106	0.554913	0.540228	0.021565
IFOF	0.456781	0.470983	0.484063	0.469909	0.020623
ILF	0.442675	0.456176	0.468337	0.454864	0.019634
ML	0.411754	0.425720	0.439422	0.425640	0.021940
CGH	0.292866	0.310008	0.325722	0.307945	0.027743
PTR	0.443471	0.457798	0.469995	0.456074	0.020733
SLF	0.421388	0.435032	0.447461	0.433901	0.020334
STR	0.409558	0.421330	0.433099	0.421671	0.018408
UF	0.372057	0.385837	0.398764	0.385260	0.019889
**MD values**					
FMA	0.000864	0.000897	0.000933	0.000902	0.000053
FMI	0.000814	0.000835	0.000858	0.000837	0.000033
MCP	0.000710	0.000752	0.000796	0.000757	0.000067
AR	0.000767	0.000786	0.000808	0.000788	0.000032
ATR	0.000763	0.000784	0.000809	0.000789	0.000038
CGC	0.000750	0.000766	0.000783	0.000767	0.000027
CST	0.000762	0.000777	0.000793	0.000778	0.000024
IFOF	0.000789	0.000808	0.000830	0.000811	0.000033
ILF	0.000797	0.000816	0.000839	0.000819	0.000034
ML	0.000865	0.000890	0.000916	0.000891	0.000040
CGH	0.000853	0.000882	0.000918	0.000893	0.000061
PTR	0.000811	0.000833	0.000861	0.000840	0.000044
SLF	0.000728	0.000747	0.000767	0.000750	0.000032
STR	0.000744	0.000759	0.000777	0.000762	0.000029
UF	0.000782	0.000802	0.000825	0.000804	0.000034
**ICVF value**					
FMA	0.581068	0.601802	0.622200	0.600615	0.032619
FMI	0.517367	0.541343	0.564636	0.540846	0.035750
MCP	0.719232	0.738317	0.757217	0.738530	0.030501
AR	0.529493	0.546909	0.564847	0.547092	0.027066
ATR	0.517556	0.537464	0.556599	0.536400	0.030661
CGC	0.532781	0.553941	0.576228	0.554389	0.032887

CST	0.668376	0.683248	0.697832	0.682580	0.023045
IFOF	0.503099	0.525971	0.546692	0.524554	0.033231
ILF	0.498247	0.519068	0.538821	0.517845	0.031809
ML	0.592557	0.605811	0.619703	0.606537	0.020779
CGH	0.437365	0.452149	0.467780	0.452719	0.024926
PTR	0.498422	0.518784	0.538815	0.517817	0.031458
SLF	0.594845	0.618103	0.640148	0.616283	0.035337
STR	0.587536	0.604357	0.621882	0.603609	0.027430
UF	0.453991	0.471704	0.488873	0.471519	0.027033

Abbreviations: SD, standard deviation.

a The abbreviations of the brain phenotypes were shown in Table S2.

Table S10. Associations of dietary n-6 PUFA intake with phenotypes of gray matter by sex

Phenotypes[a]	*β* (95% CI)[b]	*P* value[c]	Sex		*P* for interaction[d]
			Male	Female	
**Subcortical phenotypes**					
NAc	-0.034 (-0.070, 0.002)	0.061	-0.034 (-0.081, 0.013)	-0.034 (-0.079, 0.011)	0.990
AMYG	-0.037 (-0.074, 0.000)	0.053	-0.041 (-0.089, 0.008)	-0.033 (-0.080, 0.014)	0.800
CN	-0.008 (-0.045, 0.029)	0.660	0.013 (-0.036, 0.061)	-0.027 (-0.073, 0.019)	0.188
HIP	-0.061 (-0.098, -0.025)	0.001	-0.088 (-0.135, -0.040)	-0.037 (-0.083, 0.008)	0.092
GP	-0.028 (-0.065, 0.008)	0.131	-0.027 (-0.074, 0.021)	-0.030 (-0.076, 0.016)	0.920
PUT	-0.025 (-0.059, 0.008)	0.141	-0.008 (-0.052, 0.036)	-0.041 (-0.083, 0.001)	0.228
THA	-0.071 (-0.105, -0.037)	<0.001	-0.069 (-0.113, -0.025)	-0.073 (-0.115, -0.031)	0.871
**Cortical phenotypes**					
**Frontal lobe**					
cACC	-0.013 (-0.052, 0.025)	0.492	-0.050 (-0.100, 0.001)	0.019 (-0.029, 0.067)	0.029
cMFG	-0.024 (-0.061, 0.013)	0.209	-0.039 (-0.087, 0.009)	-0.010 (-0.056, 0.036)	0.339
FP	-0.027 (-0.064, 0.011)	0.161	-0.051 (-0.100, -0.002)	-0.005 (-0.052, 0.042)	0.131
lOFC	-0.039 (-0.074, -0.005)	0.026	-0.042 (-0.088, 0.003)	-0.036 (-0.080, 0.007)	0.835
mOFC	-0.039 (-0.073, -0.005)	0.025	-0.050 (-0.095, -0.006)	-0.029 (-0.072, 0.014)	0.444
PCL	-0.041 (-0.079, -0.004)	0.028	-0.042 (-0.090, 0.007)	-0.041 (-0.088, 0.005)	0.994
OP	-0.033 (-0.069, 0.004)	0.080	-0.039 (-0.087, 0.009)	-0.027 (-0.074, 0.019)	0.709
ORB	-0.052 (-0.088, -0.017)	0.004	-0.054 (-0.101, -0.008)	-0.050 (-0.095, -0.005)	0.877
TRI	-0.040 (-0.076, -0.003)	0.032	-0.048 (-0.095, -0.001)	-0.032 (-0.077, 0.013)	0.585
PCG	-0.060 (-0.095, -0.025)	0.001	-0.053 (-0.099, -0.007)	-0.067 (-0.111, -0.022)	0.636
rACC	-0.016 (-0.052, 0.021)	0.393	-0.042 (-0.089, 0.006)	0.007 (-0.038, 0.053)	0.098
RMFG	-0.029 (-0.064, 0.005)	0.090	-0.032 (-0.076, 0.013)	-0.028 (-0.070, 0.015)	0.887
SFG	-0.046 (-0.081, -0.012)	0.008	-0.053 (-0.097, -0.008)	-0.041 (-0.084, 0.002)	0.678
**Temporal lobe**					
Bankssts	-0.047 (-0.084, -0.010)	0.012	-0.067 (-0.115, -0.018)	-0.030 (-0.076, 0.016)	0.223
EC	-0.041 (-0.079, -0.003)	0.033	-0.067 (-0.117, -0.018)	-0.017 (-0.065, 0.030)	0.104
FG	-0.023 (-0.058, 0.011)	0.183	-0.026 (-0.071, 0.019)	-0.021 (-0.064, 0.022)	0.848
ITG	-0.036 (-0.071, -0.002)	0.038	-0.027 (-0.071, 0.018)	-0.045 (-0.088, -0.002)	0.509
MTG	-0.035 (-0.069, -0.001)	0.043	-0.042 (-0.087, 0.003)	-0.029 (-0.072, 0.014)	0.642
PHG	-0.038 (-0.077, 0.000)	0.050	-0.046 (-0.097, 0.004)	-0.031 (-0.079, 0.017)	0.628
STG	-0.042 (-0.077, -0.007)	0.018	-0.064 (-0.110, -0.019)	-0.022 (-0.066, 0.022)	0.139
TTG	-0.040 (-0.078, -0.003)	0.037	-0.062 (-0.111, -0.012)	-0.021 (-0.069, 0.026)	0.192
**Parietal lobe**					
IPL	-0.037 (-0.073, -0.002)	0.040	-0.042 (-0.089, 0.004)	-0.033 (-0.077, 0.012)	0.743
Isthmus	-0.011 (-0.046, 0.024)	0.544	-0.020 (-0.066, 0.026)	-0.003 (-0.047, 0.041)	0.556
PoCG	-0.048 (-0.085, -0.012)	0.009	-0.050 (-0.097, -0.003)	-0.047 (-0.093, -0.002)	0.930
PCC	-0.041 (-0.077, -0.005)	0.027	-0.077 (-0.125, -0.030)	-0.008 (-0.054, 0.037)	0.020
PCUN	-0.050 (-0.085, -0.015)	0.005	-0.054 (-0.100, -0.008)	-0.047 (-0.091, -0.003)	0.793
SPL	-0.051 (-0.087, -0.014)	0.006	-0.060 (-0.108, -0.013)	-0.042 (-0.088, 0.004)	0.539
SMG	-0.055 (-0.091, -0.020)	0.002	-0.072 (-0.118, -0.026)	-0.041 (-0.085, 0.004)	0.279
**Occipital lobe**					
CUN	-0.003 (-0.039, 0.033)	0.870	0.010 (-0.038, 0.058)	-0.015 (-0.060, 0.031)	0.404
LOC	-0.029 (-0.063, 0.005)	0.090	-0.020 (-0.064, 0.025)	-0.038 (-0.081, 0.005)	0.506
LG	-0.034 (-0.071, 0.003)	0.073	-0.037 (-0.086, 0.011)	-0.031 (-0.077, 0.016)	0.820
PCAL	-0.006 (-0.043, 0.032)	0.757	-0.009 (-0.058, 0.040)	-0.003 (-0.050, 0.044)	0.858
**Insular lobe**					
INS	-0.034 (-0.068, -0.001)	0.046	-0.038 (-0.082, 0.006)	-0.031 (-0.074, 0.011)	0.801

a Dietary n-6 PUFA intake was categorized into low and high level by median, with high intake as reference. **b** A low '*P* value' (typically less than 0.05) suggests statistical significance. **c** A low '*P* for interaction ' (typically less than 0.05) suggests statistical significance.

Table S11. Associations of dietary n-6 PUFA intake with phenotypes of white matter by sex

Phenotypes	*β* (95% CI)	*P* value	Sex		*P* for interaction
			Male	Female	
**FA values**					
FMA	-0.006 (-0.044, 0.033)	0.778	-0.001 (-0.052, 0.050)	-0.009 (-0.058, 0.039)	0.795
FMI	-0.017 (-0.055, 0.021)	0.379	-0.040 (-0.090, 0.009)	0.004 (-0.044, 0.051)	0.153
MCP	-0.027 (-0.065, 0.012)	0.172	-0.027 (-0.077, 0.023)	-0.026 (-0.075, 0.022)	0.980
AR	-0.012 (-0.050, 0.027)	0.551	-0.029 (-0.079, 0.022)	0.003 (-0.045, 0.052)	0.314
ATR	-0.052 (-0.090, -0.014)	0.007	-0.071 (-0.121, -0.021)	-0.035 (-0.083, 0.013)	0.244
CGC	-0.039 (-0.077, -0.001)	0.044	-0.057 (-0.107, -0.007)	-0.023 (-0.071, 0.025)	0.270
CST	-0.036 (-0.075, 0.002)	0.061	-0.034 (-0.084, 0.016)	-0.039 (-0.087, 0.009)	0.863
IFOF	-0.029 (-0.067, 0.009)	0.140	-0.068 (-0.118, -0.018)	0.007 (-0.041, 0.055)	0.017
ILF	-0.033 (-0.071, 0.005)	0.091	-0.071 (-0.120, -0.021)	0.002 (-0.046, 0.049)	0.019
ML	-0.044 (-0.082, -0.006)	0.025	-0.038 (-0.088, 0.012)	-0.049 (-0.097, -0.001)	0.719
CGH	-0.024 (-0.063, 0.014)	0.211	-0.027 (-0.077, 0.023)	-0.022 (-0.070, 0.026)	0.857
PTR	-0.031 (-0.069, 0.007)	0.110	-0.046 (-0.095, 0.003)	-0.017 (-0.065, 0.030)	0.349
SLF	-0.029 (-0.068, 0.009)	0.132	-0.049 (-0.099, 0.001)	-0.012 (-0.060, 0.036)	0.236
STR	-0.018 (-0.057, 0.021)	0.361	-0.028 (-0.079, 0.022)	-0.009 (-0.057, 0.040)	0.543
UF	-0.029 (-0.067, 0.009)	0.141	-0.051 (-0.101, -0.002)	-0.008 (-0.056, 0.040)	0.165
**MD values**					
FMA	-0.012 (-0.051, 0.028)	0.562	0.001 (-0.050, 0.052)	-0.023 (-0.072, 0.027)	0.467
FMI	-0.001 (-0.038, 0.037)	0.972	0.018 (-0.031, 0.068)	-0.018 (-0.065, 0.030)	0.240
MCP	0.025 (-0.013, 0.063)	0.198	0.032 (-0.018, 0.081)	0.019 (-0.029, 0.066)	0.666
AR	-0.011 (-0.049, 0.028)	0.593	0.021 (-0.030, 0.072)	-0.039 (-0.088, 0.009)	0.056
ATR	0.040 (0.005, 0.075)	0.024	0.055 (0.009, 0.100)	0.027 (-0.017, 0.071)	0.336
CGC	0.004 (-0.035, 0.042)	0.852	0.019 (-0.031, 0.070)	-0.010 (-0.059, 0.038)	0.354
CST	-0.023 (-0.061, 0.016)	0.252	-0.010 (-0.06, 0.041)	-0.034 (-0.083, 0.014)	0.435
IFOF	0.030 (-0.007, 0.067)	0.109	0.062 (0.014, 0.110)	0.001 (-0.045, 0.048)	0.044
ILF	0.023 (-0.014, 0.060)	0.222	0.049 (0.001, 0.098)	0.000 (-0.047, 0.046)	0.102
ML	-0.004 (-0.044, 0.035)	0.827	0.009 (-0.042, 0.060)	-0.016 (-0.065, 0.033)	0.433
CGH	0.028 (-0.010, 0.066)	0.154	0.049 (-0.001, 0.099)	0.008 (-0.039, 0.056)	0.190
PTR	0.055 (0.019, 0.090)	0.003	0.067 (0.021, 0.114)	0.043 (-0.002, 0.088)	0.410
SLF	0.024 (-0.013, 0.061)	0.205	0.046 (-0.003, 0.094)	0.005 (-0.042, 0.051)	0.176
STR	0.027 (-0.010, 0.063)	0.149	0.039 (-0.008, 0.087)	0.016 (-0.030, 0.061)	0.430
UF	0.032 (-0.005, 0.069)	0.093	0.057 (0.009, 0.106)	0.009 (-0.038, 0.055)	0.111
**ICVF values**					
FMA	-0.005 (-0.044, 0.033)	0.790	-0.023 (-0.074, 0.027)	0.011 (-0.037, 0.059)	0.271
FMI	-0.015 (-0.053, 0.023)	0.431	-0.036 (-0.085, 0.014)	0.003 (-0.044, 0.051)	0.203
MCP	-0.017 (-0.056, 0.021)	0.372	-0.033 (-0.083, 0.017)	-0.004 (-0.052, 0.044)	0.354
AR	-0.021 (-0.059, 0.018)	0.295	-0.047 (-0.097, 0.004)	0.003 (-0.046, 0.051)	0.117
ATR	-0.035 (-0.071, 0.001)	0.060	-0.051 (-0.099, -0.004)	-0.020 (-0.066, 0.026)	0.292
CGC	-0.038 (-0.076, -0.001)	0.046	-0.073 (-0.122, -0.024)	-0.007 (-0.054, 0.040)	0.033
CST	-0.016 (-0.055, 0.022)	0.412	-0.029 (-0.08, 0.021)	-0.004 (-0.053, 0.044)	0.432
IFOF	-0.033 (-0.071, 0.004)	0.084	-0.066 (-0.115, -0.016)	-0.004 (-0.051, 0.043)	0.044
ILF	-0.030 (-0.068, 0.007)	0.115	-0.057 (-0.106, -0.007)	-0.007 (-0.054, 0.041)	0.105
ML	-0.009 (-0.047, 0.029)	0.639	-0.002 (-0.051, 0.048)	-0.016 (-0.063, 0.032)	0.644
CGH	-0.026 (-0.065, 0.012)	0.181	-0.038 (-0.088, 0.013)	-0.016 (-0.064, 0.032)	0.489
PTR	-0.028 (-0.065, 0.009)	0.141	-0.054 (-0.103, -0.005)	-0.004 (-0.051, 0.042)	0.102
SLF	-0.031 (-0.069, 0.006)	0.103	-0.052 (-0.102, -0.003)	-0.012 (-0.060, 0.035)	0.195
STR	-0.040 (-0.078, -0.003)	0.035	-0.053 (-0.102, -0.004)	-0.029 (-0.076, 0.018)	0.418
UF	-0.044 (-0.081, -0.006)	0.022	-0.070 (-0.119, -0.021)	-0.020 (-0.067, 0.027)	0.098

Table S12. Associations of dietary n-6 PUFA intake with the phenotypes of brain gray matter

Phenotypes[a]	DEM		PD		MS	
	HR	*P* value	HR	*P* value	HR	*P* value
**Subcortical phenotypes**						
NAc	0.58 (0.42, 0.79)	0.006	0.76 (0.50, 1.15)	0.553	0.66 (0.41, 1.07)	0.413
AMYG	0.66 (0.48, 0.91)	0.028	1.14 (0.76, 1.70)	0.785	0.50 (0.30, 0.82)	0.060
CN	0.82 (0.60, 1.12)	0.269	0.98 (0.65, 1.47)	0.971	0.55 (0.32, 0.91)	0.176
HIP	0.43 (0.33, 0.57)	<0.001	0.61 (0.41, 0.90)	0.240	0.62 (0.39, 0.98)	0.211
GP	0.74 (0.53, 1.02)	0.102	1.34 (0.91, 1.93)	0.504	0.47 (0.28, 0.80)	0.060
PUT	0.59 (0.43, 0.81)	0.006	0.77 (0.50, 1.16)	0.553	0.62 (0.37, 1.01)	0.300
THA	0.60 (0.43, 0.83)	0.008	0.87 (0.57, 1.31)	0.782	0.39 (0.23, 0.66)	<0.001
**Cortical phenotypes**						
**Frontal lobe**						
cACC	0.67 (0.48, 0.92)	0.029	1.01 (0.66, 1.50)	0.978	1.16 (0.72, 1.82)	0.820
cMFG	0.78 (0.56, 1.07)	0.172	0.81 (0.53, 1.23)	0.632	1.02 (0.63, 1.62)	0.974
FP	0.73 (0.53, 1.00)	0.083	0.79 (0.51, 1.20)	0.587	1.35 (0.85, 2.08)	0.594
lOFC	0.83 (0.60, 1.13)	0.289	0.74 (0.48, 1.12)	0.553	0.70 (0.42, 1.14)	0.550
mOFC	0.84 (0.61, 1.15)	0.303	0.75 (0.48, 1.13)	0.553	0.57 (0.34, 0.94)	0.207
PCL	0.91 (0.67, 1.24)	0.573	0.68 (0.43, 1.04)	0.504	0.83 (0.50, 1.35)	0.820
OP	0.79 (0.57, 1.08)	0.196	0.95 (0.62, 1.42)	0.933	1.00 (0.61, 1.58)	0.993
ORB	0.61 (0.44, 0.85)	0.011	0.57 (0.36, 0.88)	0.240	0.85 (0.52, 1.38)	0.820
TRI	0.72 (0.51, 0.99)	0.074	0.76 (0.49, 1.16)	0.553	0.93 (0.56, 1.48)	0.960
PCG	0.69 (0.50, 0.95)	0.040	0.71 (0.46, 1.07)	0.504	0.84 (0.51, 1.35)	0.820
rACC	0.60 (0.43, 0.83)	0.008	0.96 (0.63, 1.43)	0.933	0.98 (0.60, 1.57)	0.974
RMFG	0.69 (0.50, 0.96)	0.045	0.70 (0.45, 1.07)	0.504	0.87 (0.53, 1.40)	0.847
SFG	0.69 (0.49, 0.95)	0.045	0.79 (0.51, 1.20)	0.587	1.16 (0.72, 1.82)	0.820
**Temporal lobe**						
Bankssts	0.52 (0.37, 0.73)	<0.001	0.87 (0.56, 1.30)	0.782	0.66 (0.39, 1.09)	0.452
EC	0.67 (0.48, 0.93)	0.035	0.88 (0.57, 1.32)	0.786	0.45 (0.26, 0.76)	0.060
FG	0.59 (0.42, 0.81)	0.008	0.62 (0.40, 0.95)	0.440	0.93 (0.57, 1.49)	0.960
ITG	0.57 (0.41, 0.80)	0.006	1.06 (0.70, 1.58)	0.933	0.75 (0.45, 1.21)	0.711
MTG	0.55 (0.39, 0.76)	<0.001	1.04 (0.68, 1.55)	0.933	0.98 (0.60, 1.56)	0.974
PHG	0.65 (0.47, 0.89)	0.025	0.80 (0.52, 1.21)	0.604	0.80 (0.49, 1.29)	0.809
STG	0.65 (0.47, 0.90)	0.028	0.68 (0.44, 1.05)	0.504	0.96 (0.59, 1.54)	0.974
TTG	0.91 (0.66, 1.24)	0.573	1.15 (0.76, 1.69)	0.782	0.85 (0.51, 1.37)	0.820
**Parietal lobe**						
IPL	0.53 (0.38, 0.74)	<0.001	0.71 (0.46, 1.08)	0.504	0.83 (0.50, 1.34)	0.820
Isthmus	0.74 (0.53, 1.02)	0.102	1.16 (0.77, 1.70)	0.782	0.76 (0.45, 1.24)	0.760
PoCG	0.67 (0.48, 0.92)	0.032	0.95 (0.62, 1.42)	0.933	0.89 (0.54, 1.43)	0.919
PCC	0.80 (0.58, 1.09)	0.196	0.86 (0.57, 1.30)	0.782	0.92 (0.57, 1.48)	0.960
PCUN	0.67 (0.48, 0.92)	0.030	0.76 (0.49, 1.16)	0.553	0.95 (0.58, 1.51)	0.974
SPL	0.67 (0.48, 0.92)	0.029	0.65 (0.42, 1.00)	0.504	0.79 (0.48, 1.28)	0.809
SMG	0.65 (0.46, 0.90)	0.028	0.77 (0.50, 1.17)	0.553	0.69 (0.41, 1.14)	0.550
**Occipital lobe**						
CUN	0.91 (0.66, 1.24)	0.573	1.04 (0.68, 1.54)	0.933	0.95 (0.57, 1.51)	0.974
LOC	0.66 (0.47, 0.90)	0.028	0.93 (0.61, 1.39)	0.933	0.91 (0.56, 1.46)	0.960
LG	0.65 (0.46, 0.89)	0.025	0.96 (0.63, 1.44)	0.933	0.97 (0.59, 1.54)	0.974
PCAL	0.83 (0.60, 1.14)	0.293	0.99 (0.64, 1.46)	0.971	0.79 (0.46, 1.28)	0.809
**Insular lobe**						
INS	0.84 (0.61, 1.14)	0.293	0.96 (0.63, 1.43)	0.933	0.82 (0.49, 1.32)	0.820

Abbreviations: DEM, dementia; PD, Parkinson's disease; MS, multiple sclerosis; HR, hazard ratio.

Table S13. Associations of dietary n-6 PUFA intake with the phenotypes of brain white matter

Phenotypes[a]	DEM		PD		MS	
	HR	*P* value	HR	*P* value	HR	*P* value
**FA values**						
FMA	0.72 (0.55, 0.96)	0.050	0.95 (0.65, 1.45)	0.879	0.53 (0.36, 0.80)	0.030
FMI	0.68 (0.50, 0.91)	0.025	0.66 (0.45, 0.99)	0.331	0.71 (0.45, 1.13)	0.459
MCP	0.96 (0.71, 1.30)	0.833	0.92 (0.61, 1.38)	0.812	0.69 (0.44, 1.10)	0.438
AR	1.02 (0.75, 1.39)	0.927	1.05 (0.70, 1.58)	0.879	0.72 (0.45, 1.16)	0.467
ATR	0.65 (0.48, 0.87)	0.012	0.67 (0.45, 1.00)	0.331	0.79 (0.50, 1.26)	0.598
CGC	0.59 (0.44, 0.80)	0.004	0.73 (0.49, 1.10)	0.408	0.75 (0.47, 1.21)	0.525
CST	0.92 (0.68, 1.25)	0.680	1.28 (0.85, 1.96)	0.463	0.75 (0.48, 1.20)	0.520
IFOF	0.64 (0.48, 0.86)	0.011	0.64 (0.43, 0.95)	0.331	0.52 (0.33, 0.81)	0.043
ILF	0.72 (0.54, 0.96)	0.051	0.67 (0.46, 0.99)	0.331	0.44 (0.29, 0.68)	<0.001
ML	1.04 (0.77, 1.42)	0.833	0.95 (0.63, 1.42)	0.879	0.76 (0.47, 1.22)	0.535
CGH	0.74 (0.56, 0.99)	0.069	1.29 (0.85, 1.99)	0.460	0.60 (0.40, 0.93)	0.098
PTR	0.72 (0.54, 0.97)	0.060	0.68 (0.46, 1.00)	0.331	0.37 (0.24, 0.56)	<0.001
SLF	0.66 (0.50, 0.89)	0.016	0.75 (0.51, 1.12)	0.408	0.83 (0.53, 1.34)	0.657
STR	0.83 (0.60, 1.12)	0.315	1.31 (0.88, 1.94)	0.414	0.75 (0.46, 1.22)	0.535
UF	0.86 (0.63, 1.17)	0.443	0.67 (0.44, 1.00)	0.331	0.92 (0.58, 1.49)	0.887
**MD values**						
FMA	1.38 (1.03, 1.80)	0.051	0.73 (0.46, 1.12)	0.408	1.29 (0.81, 1.96)	0.537
FMI	1.35 (1.01, 1.81)	0.075	1.27 (0.85, 1.88)	0.458	1.37 (0.86, 2.14)	0.467
MCP	1.60 (1.21, 2.08)	0.004	1.41 (0.96, 2.02)	0.398	1.56 (1.00, 2.34)	0.200
AR	1.13 (0.83, 1.52)	0.528	1.14 (0.76, 1.68)	0.710	1.52 (0.97, 2.35)	0.278
ATR	1.75 (1.36, 2.23)	<0.001	1.43 (0.98, 2.01)	0.331	1.26 (0.79, 1.90)	0.596
CGC	1.41 (1.04, 1.89)	0.050	1.11 (0.74, 1.66)	0.751	1.02 (0.63, 1.63)	0.988
CST	1.48 (1.10, 1.97)	0.023	1.02 (0.67, 1.52)	0.954	1.36 (0.85, 2.14)	0.467
IFOF	1.68 (1.27, 2.19)	<0.001	1.27 (0.85, 1.84)	0.458	1.78 (1.16, 2.66)	0.053
ILF	1.69 (1.29, 2.21)	<0.001	1.29 (0.87, 1.88)	0.421	1.72 (1.12, 2.58)	0.075
ML	1.29 (0.95, 1.74)	0.153	1.14 (0.76, 1.71)	0.710	1.27 (0.79, 2.02)	0.598
CGH	1.87 (1.47, 2.35)	<0.001	1.31 (0.89, 1.85)	0.408	1.33 (0.85, 1.97)	0.467
PTR	1.65 (1.27, 2.12)	<0.001	1.32 (0.89, 1.87)	0.408	1.76 (1.17, 2.54)	0.043
SLF	1.69 (1.30, 2.16)	<0.001	1.31 (0.89, 1.87)	0.408	1.34 (0.85, 2.01)	0.467
STR	1.85 (1.41, 2.41)	<0.001	1.37 (0.92, 1.99)	0.408	1.23 (0.77, 1.91)	0.598
UF	1.59 (1.19, 2.10)	0.004	1.39 (0.93, 2.02)	0.408	0.88 (0.54, 1.42)	0.769
**ICVF values**						
FMA	0.59 (0.44, 0.78)	<0.001	0.99 (0.66, 1.50)	0.974	0.43 (0.28, 0.66)	<0.001
FMI	0.73 (0.54, 0.99)	0.075	0.84 (0.56, 1.26)	0.644	0.70 (0.44, 1.13)	0.459
MCP	0.98 (0.72, 1.34)	0.946	1.13 (0.75, 1.70)	0.714	0.61 (0.38, 0.99)	0.211
AR	0.74 (0.54, 1.00)	0.085	0.92 (0.61, 1.39)	0.812	0.80 (0.50, 1.29)	0.598
ATR	0.65 (0.48, 0.87)	0.012	0.73 (0.49, 1.09)	0.408	0.74 (0.47, 1.19)	0.488
CGC	0.67 (0.49, 0.92)	0.028	0.86 (0.57, 1.29)	0.693	0.88 (0.55, 1.42)	0.769
CST	0.78 (0.58, 1.05)	0.152	1.13 (0.75, 1.71)	0.731	0.58 (0.37, 0.91)	0.098
IFOF	0.64 (0.48, 0.87)	0.012	0.78 (0.52, 1.17)	0.458	0.53 (0.33, 0.84)	0.050
ILF	0.64 (0.48, 0.86)	0.011	0.83 (0.56, 1.25)	0.608	0.52 (0.33, 0.82)	0.047
ML	1.01 (0.74, 1.37)	0.959	1.08 (0.72, 1.62)	0.819	0.70 (0.43, 1.14)	0.462
CGH	0.59 (0.43, 0.80)	0.004	1.25 (0.83, 1.87)	0.505	0.82 (0.51, 1.32)	0.631
PTR	0.68 (0.51, 0.92)	0.027	0.85 (0.57, 1.28)	0.675	0.43 (0.27, 0.67)	0.000
SLF	0.63 (0.48, 0.85)	0.008	0.81 (0.55, 1.21)	0.513	0.72 (0.46, 1.14)	0.459
STR	0.61 (0.46, 0.82)	0.004	0.76 (0.52, 1.14)	0.408	0.66 (0.43, 1.05)	0.313
UF	0.73 (0.54, 0.99)	0.075	0.82 (0.54, 1.23)	0.559	0.99 (0.62, 1.60)	0.997

Abbreviations: DEM, dementia; PD, Parkinson's disease; MS, multiple sclerosis; HR, hazard ratio.

Table S14. Sensitivity analysis of the main associations

Sensitivity analysis[a]	Dietary n-6 PUFA intake		*P* value
	High	Low	
**DEM**			
Sensitivity analysis 1	Reference	1.31 (1.14, 1.51)	<0.001
Sensitivity analysis 2	Reference	1.31 (1.14, 1.50)	<0.001
Sensitivity analysis 3	Reference	1.28 (1.11, 1.47)	0.001
Sensitivity analysis 4	Reference	1.35 (1.14, 1.61)	0.001
Sensitivity analysis 5	Reference	1.28 (1.10, 1.48)	0.001
**PD**			
Sensitivity analysis 1	Reference	1.43 (1.16, 1.75)	0.001
Sensitivity analysis 2	Reference	1.43 (1.16, 1.75)	0.001
Sensitivity analysis 3	Reference	1.43 (1.16, 1.75)	0.001
Sensitivity analysis 4	Reference	1.48 (1.17, 1.88)	0.001
Sensitivity analysis 5	Reference	1.42 (1.15, 1.76)	0.001
**MS**			
Sensitivity analysis 1	Reference	1.75 (1.09, 2.83)	0.022
Sensitivity analysis 2	Reference	1.66 (1.03, 2.67)	0.038
Sensitivity analysis 3	Reference	1.68 (1.00, 2.82)	0.049
Sensitivity analysis 4	Reference	1.69 (1.03, 2.76)	0.037
Sensitivity analysis 5	Reference	1.64 (1.00, 2.69)	0.049

Abbreviations: DEM, dementia; PD, Parkinson's disease; MS, multiple sclerosis.

a Sensitivity analysis 1: restricting participants to individuals with White European ancestry; Sensitivity analysis 2: controlling for various environmental factors (proximity to roadways, noise pollution, nitrogen oxide, and fine particulate matter [PM2.5]) that could potentially influence the outcomes; Sensitivity analysis 3: excluding participants who had been diagnosed with a targeted disease event within the first two years of follow-up; Sensitivity analysis 4: advancing the deadline for follow-up to December 31, 2019, in light of the potential impact of the COVID-19 outbreak on findings; Sensitivity analysis 5: additionally adjusting for income and education in the models, despite already accounting for IMD, to address potential residual confounding related to socioeconomic status.
